# Clinical setting-dependent diagnostic accuracy of artificial intelligence and store-and-forward diabetic retinopathy screening: a systematic review and meta-analysis

**DOI:** 10.1038/s41746-026-02627-0

**Published:** 2026-05-15

**Authors:** Kai-Yang Chen, Hoi-Chun Chan, Chi-Ming Chan

**Affiliations:** 1 Department of General Medicine, Chang Gung Memorial Hospital (Linkou branch), Taoyuan, Taiwan; 2 School of Pharmacy, China Medical University, Taichung, Taiwan; 3 Department of Ophthalmology, Cardinal Tien Hospital, New Taipei City, Taiwan; 4 School of Medicine, Fu Jen Catholic University, New Taipei City, Taiwan

**Keywords:** Computational biology and bioinformatics, Diseases, Health care, Mathematics and computing, Medical research

## Abstract

Population-based diabetic retinopathy (DR) screening requires diagnostic strategies that optimize clinical utility by balancing missed disease against referral burden. We performed a Preferred Reporting Items for Systematic Reviews and Meta-Analyses of Diagnostic Test Accuracy studies (PRISMA-DTA)-guided systematic review and meta-analysis comparing autonomous artificial intelligence (AI) screening with store-and-forward (SAF) or conventional image-based teleophthalmology pathways, using manual, expert, or reading-center grading as the reference standard, across any DR, referable DR (RDR), vision-threatening DR (VTDR), and diabetic macular edema (DME). Twenty-eight diagnostic accuracy studies were included. AI showed higher pooled sensitivity than SAF for any DR (86.9% vs 80.9%), RDR (96.2% vs 88.6%), VTDR (96.2% vs 84.2%), and DME (97.2% vs 87.4%). AI also showed higher pooled specificity for any DR, RDR, and VTDR, whereas DME specificity was similar between pathways. Translating operating characteristics into decision consequences demonstrated that pathway preference depends on prevalence, decision thresholds, and misclassification weighting: at 15% prevalence, AI yielded higher net benefit (140.7 vs 120.8 net true-positive decisions per 1000 screened at pₜ = 0.10). These findings support pathway-specific deployment strategies rather than direct superiority claims.

## Introduction

Diabetic retinopathy (DR) remains a leading cause of preventable vision loss among working-age adults worldwide, and it represents a major public health and health-system equity challenge in the United States, where the expanding diabetes population, fragmented screening uptake, and regional workforce shortages continue to drive delayed diagnosis and avoidable vision-threatening complications. With the global burden of diabetes steadily increasing, the demand for accurate and scalable DR screening has intensified. Routine surveillance and early detection of retinal lesions are essential to prevent irreversible visual impairment, yet structural barriers-including geographic maldistribution of eye-care providers and limited screening capacity in rural and underserved communities-continue to restrict timely access to in-person ophthalmic evaluation. Recent global estimates suggest that over 93 million people are affected by DR, with many more undiagnosed due to inadequate screening access, especially in low- and middle-income countries^1^. Teleophthalmology is increasingly recognized as a viable means to expand early detection efforts in line with WHO goals for universal eye health coverage^2,3^.

Teleophthalmology has emerged to bridge this gap by combining digital fundus photography with remote image review^4^. Two principal models have gained traction: SAF workflows using remote interpretation by human graders and algorithm-driven AI platforms enabling automated image assessment at scale. In the former, retinal images captured at satellite sites are transmitted to specialist readers, a process that has demonstrated high diagnostic reliability under standardized protocols and trained operators^5^.

In rural settings, store-and-forward workflows have enabled DR screening by trained non-specialist personnel, improving reach without sacrificing diagnostic integrity^6^; similar programs in Latin America have shown sustainable scalability^7^.

In algorithm-driven frameworks, machine learning tools such as EyeArt and IDx-DR perform automated image interpretation, demonstrating diagnostic performance comparable to reference grading in selected validation settings and obtaining regulatory clearance in selected jurisdictions and supporting deployment in defined clinical-use scenarios^8,9^. In the United States, autonomous AI DR screening has marked a regulatory and implementation milestone, enabling point-of-care deployment in primary-care workflows without requiring immediate ophthalmologist overread^10^.

These systems have demonstrated over 90% sensitivity in prospective studies, with scalability that supports large-scale national screening programs^11^; in Spain, the IDx-DR algorithm showed 100% sensitivity for referable DR^12^.

A growing body of research confirms that teleophthalmology attains sensitivity and specificity values approaching those of traditional fundus photography and face-to-face assessment^13,14^. Economic evaluations further underscore its value: a Brazilian analysis reported that a teleophthalmology model based on fundus imaging was both clinically effective and more cost-efficient per patient than conventional screening^7^, while an Indian program operated by trained nurses achieved comparable diagnostic yield with sustainable expenditure^6^.

Despite these advances, obstacles remain. Variations in image quality, heterogeneity in reader expertise, and fluctuating DR prevalence across populations can influence diagnostic performance^15^. Additionally, the need for robust IT infrastructure, secure data transfer, and seamless integration into existing healthcare workflows presents logistical and regulatory challenges^16^; user trust and model explainability also remain critical barriers to AI adoption^17^.

Algorithm-based teleophthalmology has the potential to alleviate some of these limitations by reducing reliance on human graders and enhancing throughput. Yet its generalizability across diverse settings, the transparency of model decisions, and comparative performance metrics warrant further scrutiny. Hybrid workflows where algorithmic screening is supplemented by expert overreads have shown promise in boosting both accuracy and operational efficiency^9,18^.

Although many studies report sensitivity and specificity for autonomous AI screening and SAF teleophthalmology, implementation decisions are increasingly judged by decision consequences-missed referral, relevant disease, referral burden, and the relative cost of false negatives versus false positives-rather than by metrics alone. Contemporary methodological work emphasizes that diagnostic accuracy is not equivalent to clinical utility, and recommends interpreting operating characteristics through decision thresholds, misclassification costs, and net benefit (decision curve) concepts, even when individual-level risk models are unavailable^19,20^. Importantly, autonomous AI and SAF teleophthalmology are not merely two “tests,” but two pathways with different workflows, case-mix, imaging protocols, and adjudication structures; therefore, indirect comparisons can be misleading when evidence streams are non-overlapping. The objective of this PRISMA-DTA-aligned systematic review and meta-analysis was therefore not to claim head-to-head superiority, but to generate pathway-specific operating characteristics for (1) autonomous AI screening and (2) SAF teleophthalmology (human graders), each evaluated against a shared reference standard (manual grading), for detecting any DR, RDR, VTDR, and DME. We additionally translate pooled sensitivity/specificity into per-1000 screening consequences and provide a decision-analytic interpretation (net benefit across plausible referral thresholds) to support implementation planning, consistent with modern expectations for clinically actionable evidence in digital medicine and AI-enabled diagnostics.

## Results

**Fig. 1:**
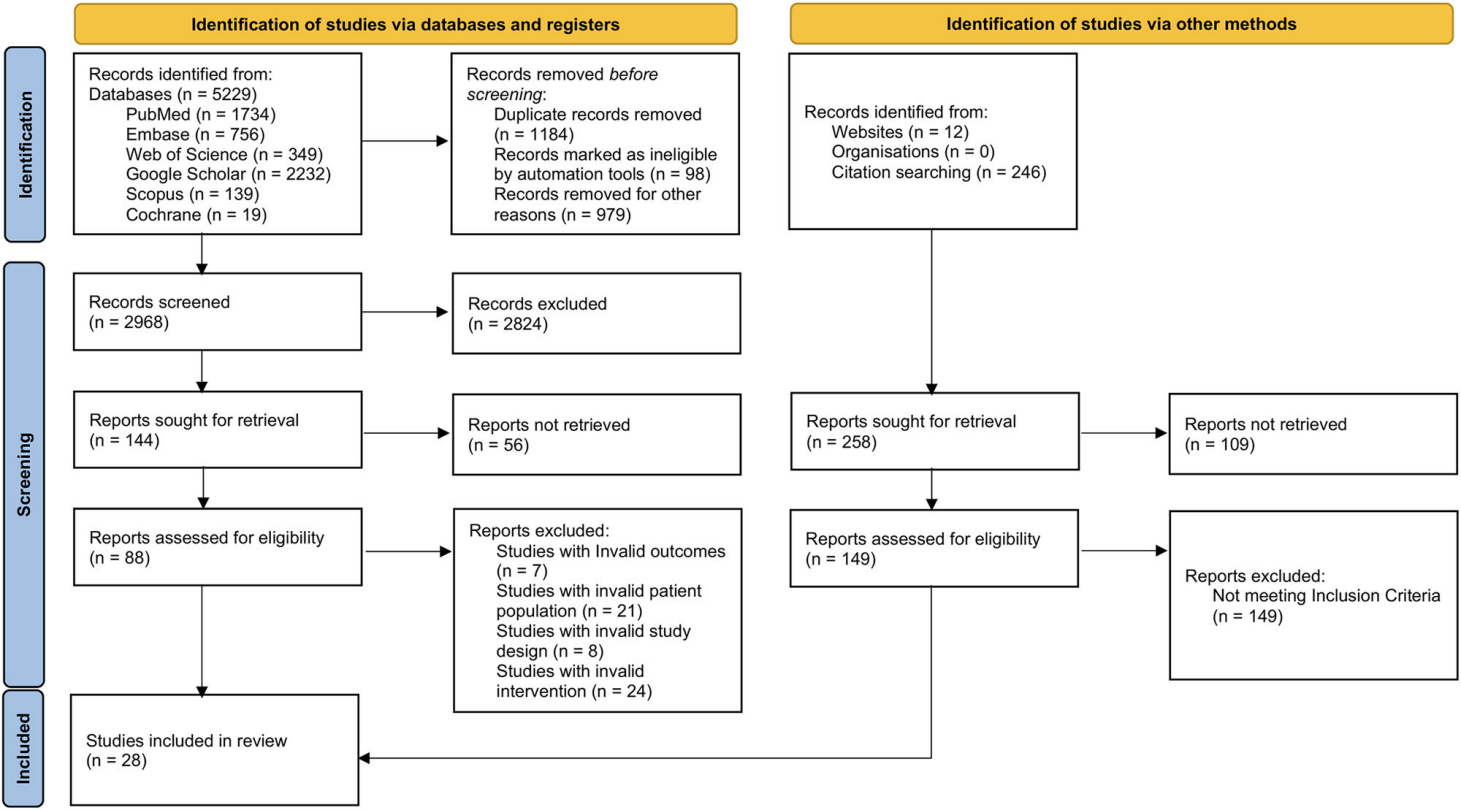
Study identification and selection process for included diagnostic accuracy studies. PRISMA 2020 flow diagram summarizing study identification from database searches and additional sources, deduplication, title and abstract screening, full-text eligibility assessment, reasons for exclusion, and final inclusion in qualitative synthesis and quantitative meta-analysis. PRISMA, Preferred Reporting Items for Systematic Reviews and Meta-Analyses.

This PRISMA-DTA-guided evidence synthesis identified 28 eligible studies evaluating the diagnostic accuracy of AI-based and SAF DR screening pathways. In addition to conventional diagnostic accuracy interpretation, pooled estimates were considered within a clinical decision-making context, recognizing that sensitivity and specificity represent operating characteristics that influence referral thresholds, false-positive burden, misclassification-cost trade-offs, and missed-disease risk in real-world screening workflows. In the database and register search pathway, 5229 records were initially identified, including 1734 from PubMed, 756 from Embase, 349 from Web of Science, 2232 from Google Scholar, 139 from Scopus, and 19 from Cochrane. Prior to screening, 2261 records were removed, comprising 1184 duplicate records, 98 records marked as ineligible by automation tools, and 979 records removed for other reasons, leaving 2968 records for title and abstract screening. Of these, 2824 records were excluded at the screening stage, resulting in 144 reports sought for retrieval, among which 56 reports were not retrieved, and 88 reports were successfully assessed for eligibility. During full-text eligibility evaluation, 60 reports were excluded due to predefined methodological or clinical ineligibility, including 7 studies with invalid outcomes, 21 studies with an invalid patient population, 8 studies with an invalid study design, and 24 studies with an invalid intervention, which led to 28 studies being included from this pathway. In parallel, the additional identification pathway via other methods yielded 258 records, including 12 from websites, 0 from organizations, and 246 from citation searching, and after retrieval attempts, 109 reports were not retrieved, leaving 149 reports assessed for eligibility; however, all 149 were excluded because they did not meet the inclusion criteria, contributing no additional eligible studies. Therefore, after integrating both pathways and applying all exclusion criteria rigorously across screening, retrieval, and eligibility stages, 28 studies met all inclusion criteria and were included in the qualitative synthesis and quantitative meta-analysis (Fig. 1). These studies formed the final evidence base for comparing diagnostic performance between AI-based and store-and-forward diabetic retinopathy screening methods.

### Study characteristics

The characteristics of the included studies are summarized in Table 1, stratified by screening modality. The studies were conducted across diverse geographic regions and clinical settings and employed a wide range of imaging protocols, grading frameworks, and AI algorithms. This heterogeneity in study design and execution was anticipated and is explicitly addressed using random-effects models, sensitivity analyses, and exploratory subgroup and meta-regression analyses.

**Table 1:** Characteristics of studies included in the primary meta-analysis

Study (Country, Year)	Design	Sample size (*n*)	DR prevalence (%)	Target condition (definition)	Imaging protocol	Index test	Reference standard (no. of graders, adjudication)	Ungradable image rate (%)
**AI-based screening studies**								
Arenas-Cavalli (Chile, 2022)^21^	Retrospective clinical validation	1123 eye exams	21.3	Any DR (R1-R4), DME suspicion, or ungradable (ICDR)	Non-mydriatic CFP; 45°; 2 fields/eye	DART (TeleDx) AI	Single ophthalmologist (panel of 8); no adjudication	1.8
Doğan (Turkey, 2024)^22^	Prospective diagnostic accuracy	865 patients	NR	mtmDR, vtDR, suspected CSDMO (AAO/ICDR)	Non-mydriatic CFP; 2 fields/eye + dilated 4-field	EyeCheckup AI	3 retina specialists; consensus	NR
Grzybowski (Poland, 2025)^23^	Retrospective comparative	750 patients (695 analyzed)	Any DR 25.0; RDR 15.3; STDR 3.5	ICDR thresholds	Non-mydriatic CFP; 4 × 45°	IDx-DR; MONA DR	3 graders; majority decision	7.3
Kubin (Finland, 2024)^24^	Prospective real-world comparison	156 patients	RDR 34.6	≥ moderate NPDR	Handheld Aurora; non-mydriatic; 8 images/patient	21 AI algorithms	2 ophthalmologists; agreement	Mean 1.9 (range 0–28.2)
Olvera-Barrios (UK, 2020)^25^	Cross-sectional screening study	1257 patients	Any DR 34.6; VTDR ~ 8	NDESP grading	Mydriatic 2-field CFP; EIDON	EyeArt v2.1	NDESP graders (levels 1–3)	NR
Sedova (Austria, 2022)^26^	Prospective pilot	54 patients	NR	≥ moderate DR or VTDR	Non-mydriatic CFP vs UWF	IDx-DR v2.2	2 retina specialists; consensus	0.9
Sosale (India, 2020)^27^	Prospective community screening	297 patients	Any DR 40.8; RDR ~ 29	ICDR; RDR ≥ moderate NPDR	Mydriatic smartphone CFP; 3 fields	Medios AI	2 VR specialists; consensus	2.3
Tang (Multinational, 2021)^28^	Retrospective + external validation	1022 subjects	NR	RDR ≥ moderate NPDR; VTDR	UWF Optos (~200°)	CNN (ResNet-50)	Retina specialists; consensus	NR
Tokuda (Japan, 2022)^29^	Prospective pilot AI	89 eyes	Any DR 23.9	Mild- or moderate-plus NPDR	Single-field CFP	DL + SVM (hemorrhage-based)	3 specialists; majority	21.3
Dow (USA, 2023)^9^	Real-world teleophthalmology	1222 encounters	MTMDR 11.9	ETDRS ≥ 35 or CSDME	Non-mydriatic CFP; 2 fields	IDx-DR	Reading-center graders + clinical exam	AI-only 36.5
Dong (China, 2022)^30^	Community screening	443 participants	Any DR 31.4; mtmDR 27.5	ICDR	Single-field CFP	CARE AI	2 ophthalmologists + adjudicator	0.9
Gulshan (India, 2019)^31^	Prospective multicentre	3049 patients	RDR ~ 23.8; VTDR ~ 10.6	ICDR/ETDRS mapping	Non-mydriatic CFP; 2 fields	Google DL v2	ETDRS graders; adjudication	NR
Hao (China, 2022)^32^	Population screening	3933 patients	Any DR 22.7	ICDR	Non-mydriatic CFP; 2 fields	EyeWisdom AI	2 ophthalmologists + chief adjudication	7.8
Jain (India, 2021)^33^	Community screening	1294 analyzed	Any DR 11.1; RDR 4.9	ICDR	Mydriatic smartphone CFP	Medios AI	2 VR surgeons + adjudicator	6.1
Kanagasingam (Australia, 2018)^34^	Primary-care deployment	193 patients	Clinically significant DR ~ 1	ICDR	Non-mydriatic CFP	DL system	Single ophthalmologist	NR
Keel (Australia, 2018)^35^	Pilot feasibility	96 patients	RDR 10.4	ICDR	Single-field CFP	EyeGrader AI	Ophthalmologist consensus	3.1
Li (China, 2021)^36^	Real-world clinic study	1147 patients	Any DR 27.9; RDR 14.6	ICDR	Non-mydriatic CFP	VoxelCloud AI	Single retinal specialist	6.6
Natarajan (India, 2019)^37^	Community screening	213 analyzed	Any DR 12.7; RDR 7.0	ICDR	Mydriatic smartphone CFP	Medios AI	VR resident + surgeon adjudication	7.8
Rajalakshmi (India, 2018)^38^	Tertiary-care validation	296 analyzed	Any DR 64.5; STDR 37.8	ICDR	Mydriatic smartphone CFP	EyeArt AI	2 retina specialists + adjudicator	1.7
Scheetz (Australia, 2021)^39^	Community screening	1061 participants	RDR ~ 7.7	ICDR	Non-mydriatic CFP; 2 fields	DL-based AI	2 ophthalmologists + adjudication	~9–10
Yang (China, 2022)^40^	Community screening	~1200 patients	Any DR ~ 25–30	ICDR	Non-mydriatic CFP	DL system	2 ophthalmologists + adjudication	~10–12
Zhang (China, 2020)^41^	Nationwide screening program	47 269 enrolled	Any DR 28.8; RDR 24.4; VTDR 10.8	ICDR	Non-mydriatic CFP; 1 field	VoxelCloud Retina	Two-stage grading + adjudication	14.0
**Store-and-forward teleophthalmology studies**								
Jacoba (Philippines/USA/UK, 2023)^42^	Prospective cross-sectional	118 patients (225 eyes)	NR	ICDR; RDR ≥ moderate NPDR or ungradable	Mydriatic; Optomed Aurora vs Optos UWF	Handheld camera	3 graders; senior adjudication	0
Lin (USA, 2002)^43^	Prospective comparative	197 patients	NR	ETDRS > 35 referral-warranted DR	Single-field 45° CFP	Telemedicine grading	ETDRS certified readers; adjudication	8.1
Rudnisky (Canada, 2007)^44^	Prospective validation	102 patients	RDR 59.8	ETDRS ≥ 61 and/or CSME	Mydriatic 7-field ETDRS	Web-based grading	2 ETDRS readers; consensus	~3
Salongcay (Philippines, 2024)^45^	Prospective instrument validation	116 patients	Any DR 66.7; RDR 46.2	ICDR; RDR ≥ moderate NPDR	Mydriatic handheld; multi-field	Handheld cameras	5 graders; central adjudication	Device-dependent (0–7.6)
Scotland (UK, 2010)^46^	Retrospective modeling	7586 cases	RDR ~ 4	Scottish DR grading	National screening CFP	Automated algorithms	Expert review; clinician adjudication	8.2
Silva (USA, 2011)^47^	Prospective comparative	67 patients	NR	ETDRS DR severity; STDR	Non-mydriatic CFP vs ETDRS film	MegaVision system	2 readers; senior adjudication	2.4

*NR* not reported, *NPDR* non-proliferative diabetic retinopathy, *PDR* proliferative DR, *DME* diabetic macular edema, *CSME* clinically significant macular edema, *ICDR* International Clinical DR scale, *ETDRS* Early Treatment Diabetic Retinopathy Study, *AAO PPP* American Academy of Ophthalmology Preferred Practice Pattern, *NDESP* National Diabetic Eye Screening Programme (UK), *UWF-SLO* Ultra-Widefield Scanning Laser Ophthalmoscope.

### Risk of bias

**Fig. 2:**
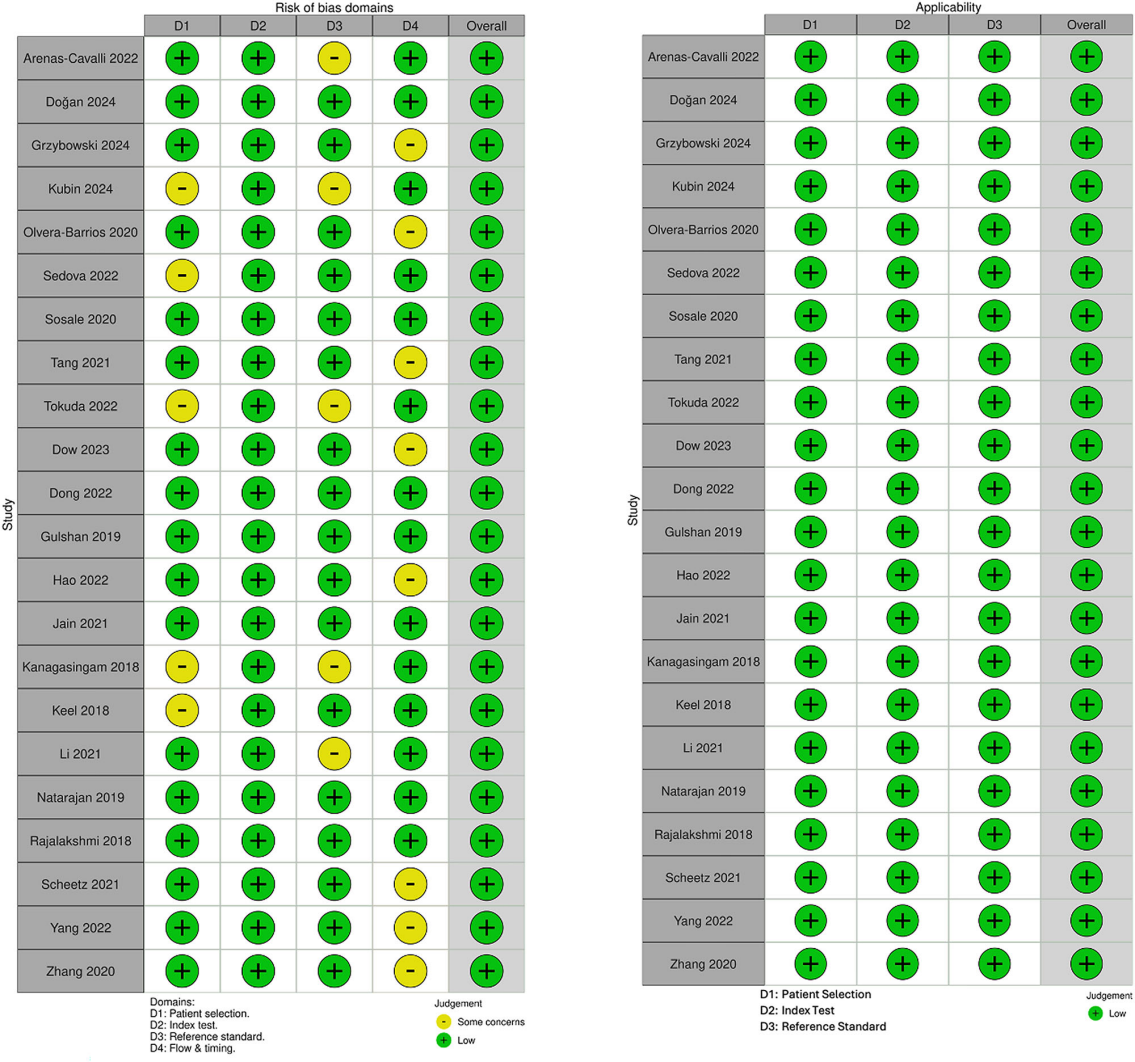
Risk of bias and applicability summary for artificial intelligence-based diabetic retinopathy screening studies. Traffic-light plot of QUADAS-2 assessments for included artificial intelligence-based diabetic retinopathy screening studies. Risk-of-bias domains include patient selection, index test, reference standard, and flow and timing. Applicability concerns include patient selection, index test, and reference standard. QUADAS-2 Quality Assessment of Diagnostic Accuracy Studies 2, AI artificial intelligence.

Figure 2 summarizes the Quality Assessment of Diagnostic Accuracy Studies 2 (QUADAS-2) traffic-light assessment for the included AI-based screening studies, showing risk-of-bias judgments across D1 patient selection, D2 index test, D3 reference standard, and D4 flow and timing, as well as applicability judgments across D1–D3 as displayed. Overall, the majority of included AI-based screening studies demonstrated a low risk of bias across all domains, indicating robust internal validity and appropriate study conduct. Most studies employed prospective or well-defined retrospective screening cohorts with minimal inappropriate exclusions, supporting a low risk in patient selection. The index-test domain consistently showed low risk, as AI algorithms were applied using pre-specified thresholds and interpreted without knowledge of reference-standard results, reducing the likelihood of test review bias. Some studies were judged as having “some concerns” in the reference-standard domain, primarily due to reliance on single graders, limited grader panels, or absence of formal adjudication processes, which may introduce classification variability. Similarly, flow and timing concerns were noted in a subset of studies where delays between index testing and reference grading occurred, or where handling of ungradable images was not consistently reported or excluded post hoc. Despite these isolated concerns, no study was judged to be at high risk of bias in any domain. Importantly, the overall risk of bias was rated as low for all included studies, reflecting that the identified concerns were unlikely to substantially distort diagnostic accuracy estimates.

**Fig. 3:**
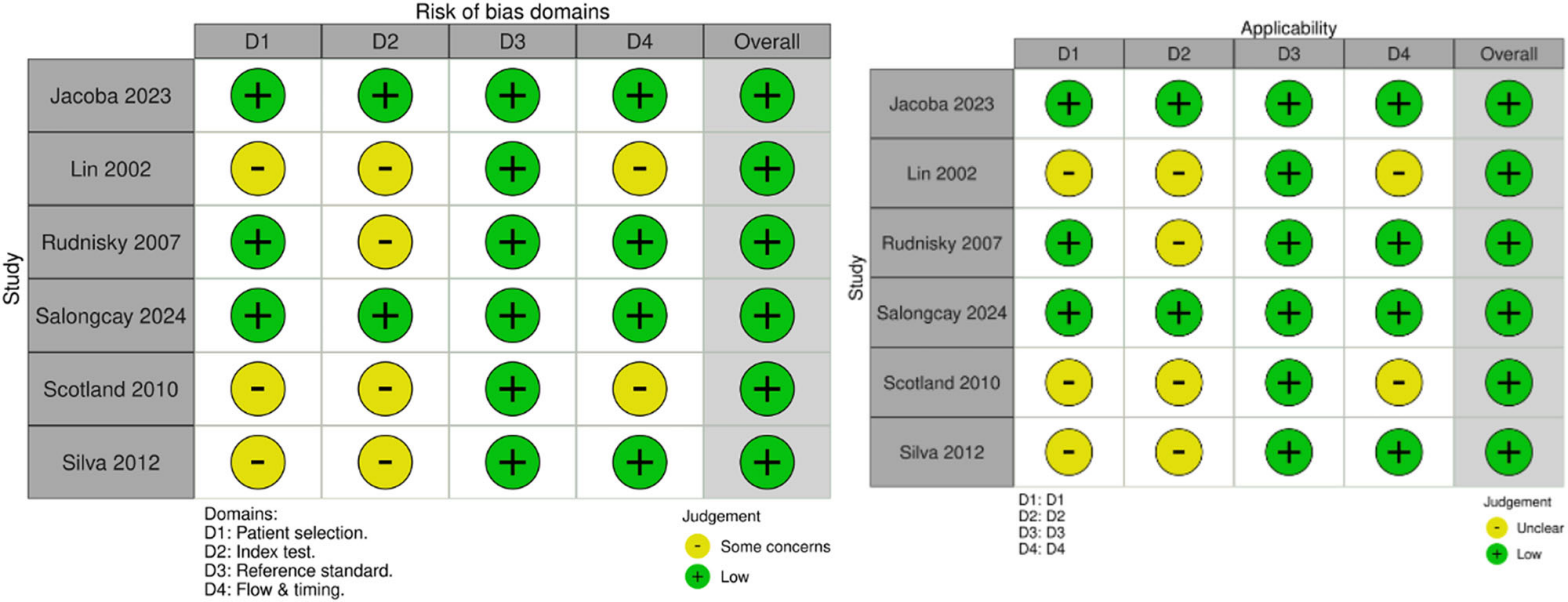
Risk of bias and applicability summary for store-and-forward teleophthalmology diabetic retinopathy screening studies. Traffic-light plot of QUADAS-2 assessments for included store-and-forward teleophthalmology diabetic retinopathy screening studies. Risk-of-bias domains include patient selection, index test, reference standard, and flow and timing. Applicability concerns include patient selection, index test, and reference standard. QUADAS-2 Quality Assessment of Diagnostic Accuracy Studies 2, SAF store-and-forward.

Figure 3 presents the QUADAS-2 traffic-light assessment for the included SAF teleophthalmology studies, reporting domain-level judgments for risk of bias and applicability according to the figure’s displayed domains. Overall, the body of evidence demonstrated a low risk of bias, with all studies ultimately judged as low risk in the overall domain, indicating acceptable methodological quality for diagnostic accuracy synthesis. Recent studies, particularly Jacoba et al.^42^ and Salongcay et al.^45^, showed consistently low risk across all domains, reflecting prospective designs, clearly defined inclusion criteria, standardized image acquisition, and use of multi-grader reference standards with adjudication. Some earlier studies, including Lin et al.^43^, Scotland et al.^46^, and Silva et al.^47^, were rated as having some concerns in patient selection and index-test domains. These concerns primarily stemmed from non-consecutive sampling, retrospective designs, older imaging protocols, and less explicit pre-specification of grading thresholds. Additionally, minor concerns in the flow and timing domain were noted where delays between image acquisition and reference grading occurred or where exclusions of ungradable images were not uniformly reported. Importantly, the reference-standard domain was consistently judged as low risk across all studies, as diagnosis was based on established grading systems including the Early Treatment Diabetic Retinopathy Study (ETDRS) scale and the International Clinical Diabetic Retinopathy (ICDR) scale, and was interpreted by trained graders, often with consensus or adjudication.

### Meta-analysis: sensitivity for any diabetic retinopathy

**Fig. 4:**
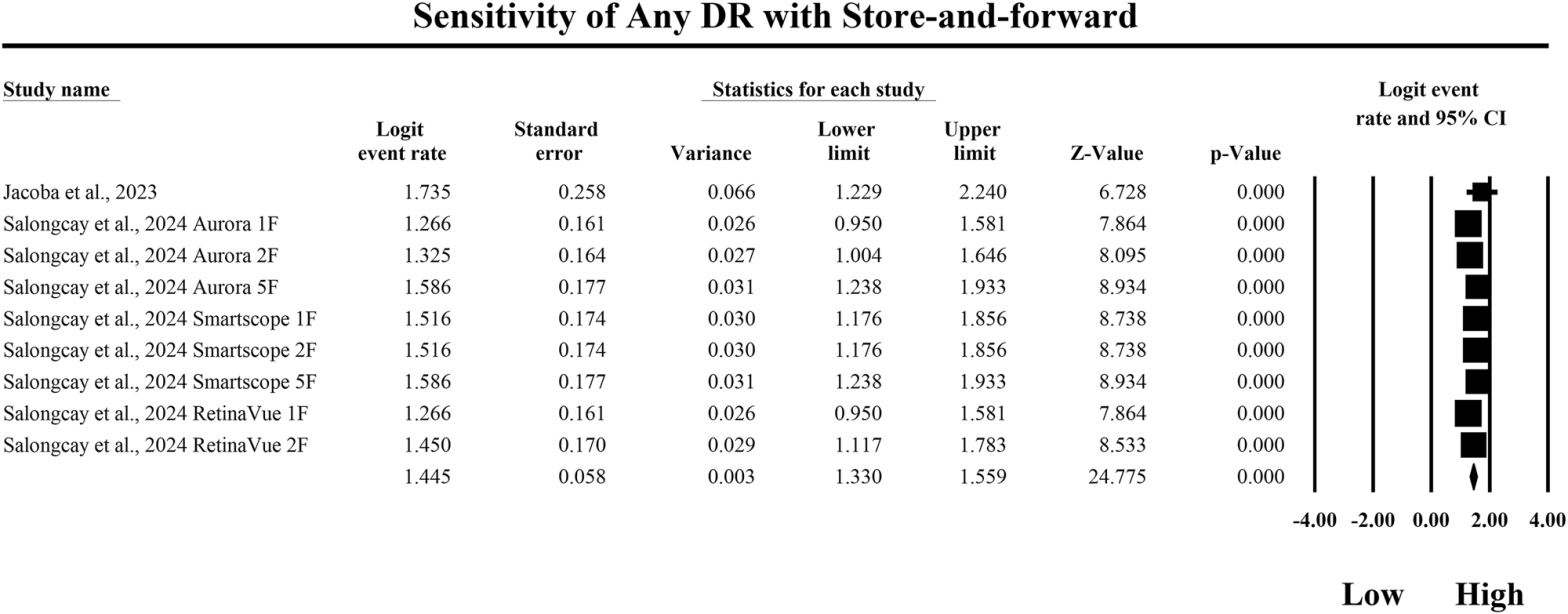
Diagnostic sensitivity of store-and-forward teleophthalmology for the detection of any diabetic retinopathy. Random-effects meta-analysis forest plot of sensitivity for detecting any diabetic retinopathy using store-and-forward teleophthalmology with human grading compared with manual reference grading. Sensitivity estimates are presented as logit-transformed event rates with 95 percent confidence intervals for individual studies and pooled effects. DR diabetic retinopathy, SAF store-and-forward, CI confidence interval.

**Fig. 5:**
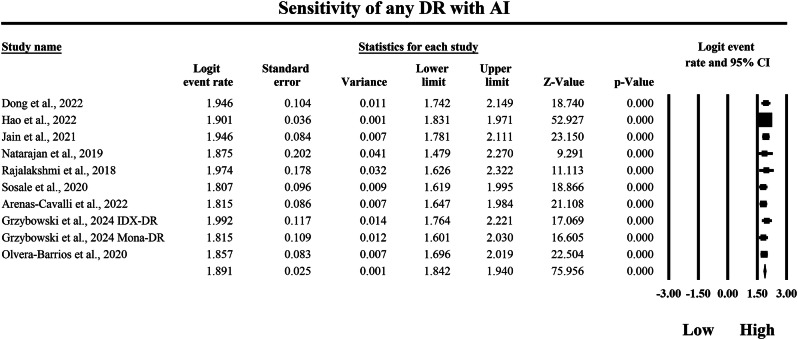
Diagnostic sensitivity of artificial intelligence-based screening for the detection of any diabetic retinopathy. Random-effects meta-analysis forest plot of sensitivity for detecting any diabetic retinopathy using artificial intelligence-based screening systems compared with manual reference grading. Sensitivity estimates are presented as logit-transformed event rates with 95 percent confidence intervals for individual studies and pooled effects. DR diabetic retinopathy, AI artificial intelligence, CI confidence interval.

The meta-analysis shown in Figs. 4 and 5 compares the pooled sensitivity of SAF teleophthalmology screening versus AI-based diagnostic systems for detecting any DR, using logit-transformed event rates with 95% confidence intervals to quantify the probability of correctly identifying true DR cases. In the SAF subgroup in Fig. 4, individual sensitivity estimates ranged from a logit event rate of 1.266 to 1.735, with study-level 95% confidence interval bounds extending from a minimum lower limit of 0.950 to a maximum upper limit of 2.240, indicating that SAF-based screening strategies generally demonstrated high sensitivity but with moderate dispersion across different screening implementations. The pooled SAF estimate at the bottom of Fig. 4 was a logit event rate of 1.445, with a 95% confidence interval from 1.330 to 1.559, reflecting consistently high overall sensitivity for detecting any DR within this screening modality. In contrast, the AI subgroup in Fig. 5 demonstrated uniformly higher sensitivity estimates, ranging from a logit event rate of 1.807 to 1.992, with study-level 95% confidence interval bounds spanning from a minimum lower limit of 1.479 to a maximum upper limit of 2.322, suggesting a tighter and more consistently elevated sensitivity profile across AI-based diagnostic systems. The pooled AI sensitivity estimate was a logit event rate of 1.891, with a 95% confidence interval from 1.842 to 1.940, which exceeds the pooled SAF estimate and therefore indicates higher pooled sensitivity within the AI evidence stream for detecting any DR in the AI subgroup. Because higher logit event rates correspond to a greater probability of correct case identification, back-transformation of the pooled logit values further supports a clinically meaningful difference between strategies, with the pooled SAF estimate corresponding to an approximate sensitivity of 80.9%, whereas the pooled AI estimate corresponds to an approximate sensitivity of 86.9%. Overall, these findings demonstrate that both SAF teleophthalmology and AI-based systems achieve high sensitivity for detecting any DR, but AI-based diagnostic approaches yield a higher pooled sensitivity estimate with a narrower pooled confidence interval, consistent with improved true-positive case detection performance across the included studies and screening configurations represented in these forest plots.

### Meta-analysis: sensitivity for referable diabetic retinopathy

**Fig. 6:**
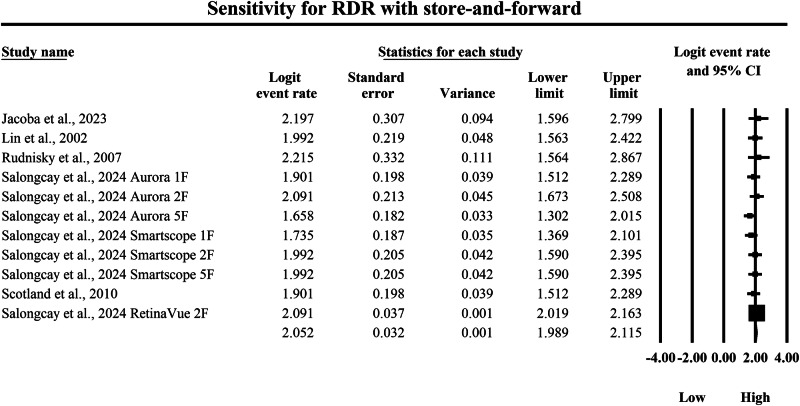
Diagnostic sensitivity of store-and-forward teleophthalmology for the detection of referable diabetic retinopathy. Random-effects meta-analysis forest plot of sensitivity for detecting referable diabetic retinopathy using store-and-forward teleophthalmology compared with manual reference grading. Sensitivity estimates are presented as logit-transformed event rates with 95 percent confidence intervals for individual studies and pooled effects. RDR referable diabetic retinopathy, SAF store-and-forward, CI confidence interval.

**Fig. 7:**
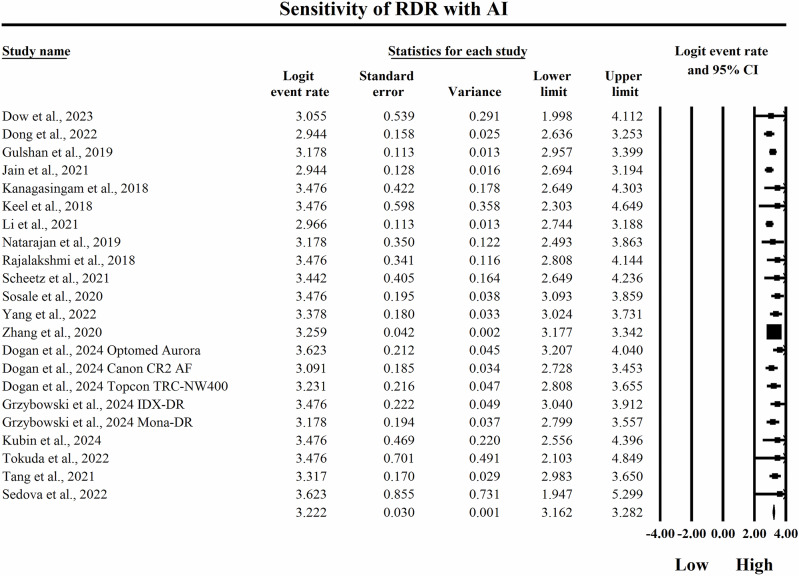
Diagnostic sensitivity of artificial intelligence-based screening for the detection of referable diabetic retinopathy. Random-effects meta-analysis forest plot of sensitivity for detecting referable diabetic retinopathy using artificial intelligence-based screening systems compared with manual reference grading. Sensitivity estimates are presented as logit-transformed event rates with 95 percent confidence intervals for individual studies and pooled effects. RDR referable diabetic retinopathy, AI artificial intelligence, CI confidence interval.

The meta-analysis presented in Figs. 6 and 7 provides a side-by-side descriptive comparison of the pooled diagnostic sensitivity for detecting RDR between SAF and AI-based diagnostic systems, with sensitivity estimates expressed as logit-transformed event rates and corresponding 95% confidence intervals. The SAF subgroup presented in Fig. 6 showed lower sensitivity estimates overall, with individual logit event rates ranging from 1.658 to 2.215, and study-specific 95% confidence interval boundaries extending from a lowest lower limit of 1.302 to a highest upper limit of 2.867, reflecting high but comparatively less elevated sensitivity for identifying RDR in SAF teleophthalmology workflows across different screening implementations. The pooled sensitivity estimate for SAF was a logit event rate of 2.052, with a 95% confidence interval from 1.989 to 2.115, which is numerically lower than the pooled AI estimate on the same logit scale and therefore indicates a lower overall probability of detecting true RDR cases within this descriptive comparison. In contrast, in the AI subgroup shown in Fig. 7, individual study sensitivity estimates were consistently high, with logit event rates ranging from 2.944 to 3.623, and study-specific 95% confidence interval boundaries spanning from a lowest lower limit of 1.947 to a highest upper limit of 5.299, indicating that AI systems generally achieved strong detection capability for RDR across included datasets while still exhibiting variability in precision among studies. The pooled sensitivity estimate for AI was a logit event rate of 3.222, with a 95% confidence interval from 3.162 to 3.282, demonstrating a very high overall probability of correctly identifying RDR cases within the AI-based screening modality. Because higher logit event rates correspond to greater sensitivity, back-transformation of the pooled estimates further supports a clinically meaningful difference between strategies, with the pooled SAF estimate corresponding to an approximate sensitivity of 88.6%, whereas the pooled AI estimate corresponds to an approximate sensitivity of 96.2%, indicating that AI-based diagnostic systems achieve substantially higher pooled sensitivity for detecting referable disease and, consequently, a lower likelihood of missed clinically significant RDR cases compared with SAF approaches within the set of studies summarized in these forest plots.

### Meta-analysis: sensitivity for vision-threatening diabetic retinopathy

**Fig. 8:**
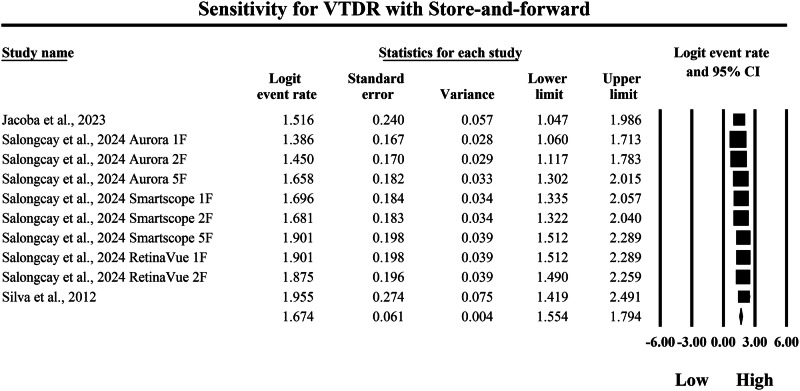
Diagnostic sensitivity of store-and-forward teleophthalmology for the detection of vision-threatening diabetic retinopathy. Random-effects meta-analysis forest plot of sensitivity for detecting vision-threatening diabetic retinopathy using store-and-forward teleophthalmology with human grading compared with manual reference grading. Sensitivity estimates are presented as logit-transformed event rates with 95 percent confidence intervals for individual studies and pooled effects. VTDR vision-threatening diabetic retinopathy, SAF store-and-forward, CI confidence interval.

**Fig. 9:**
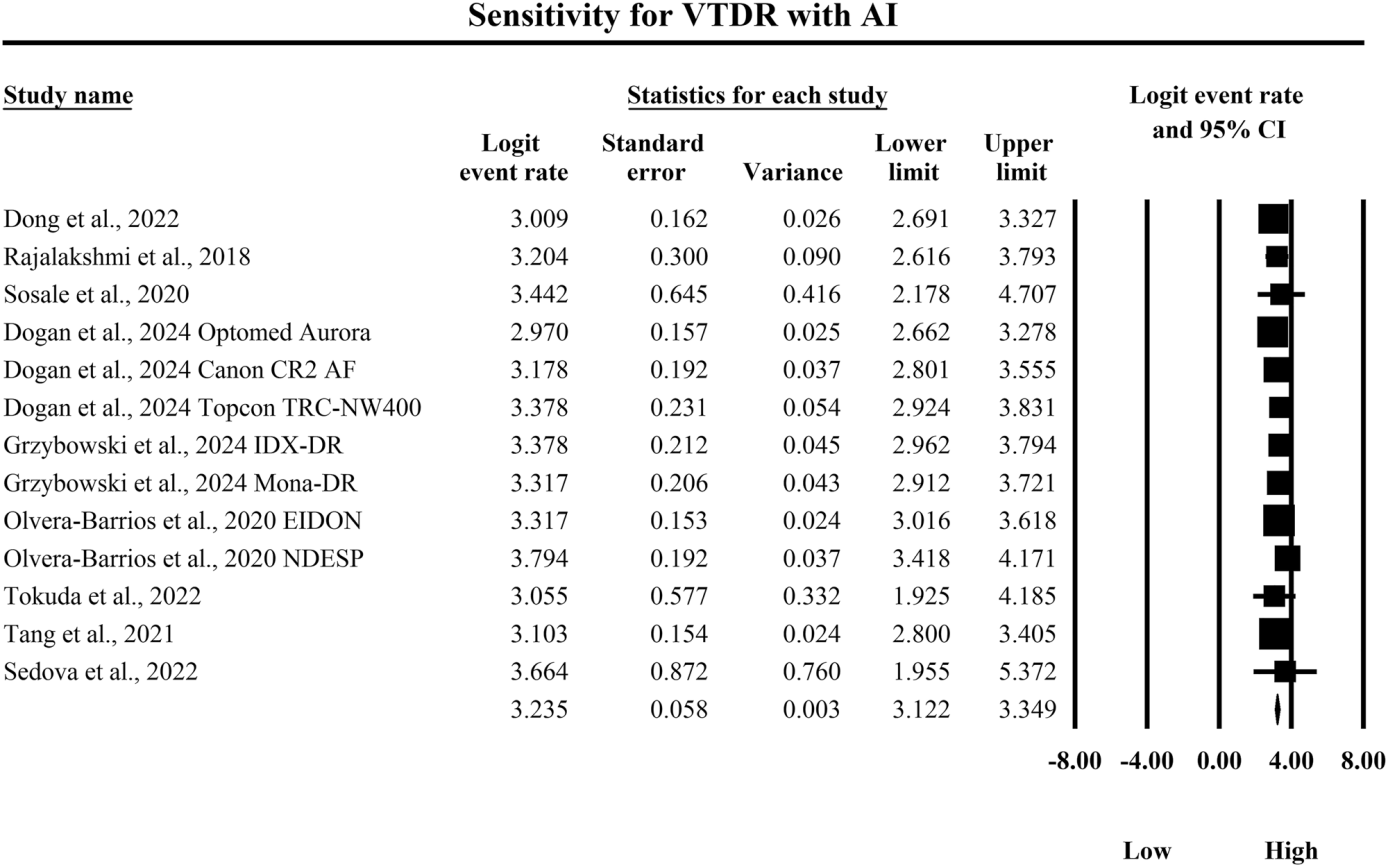
Diagnostic sensitivity of artificial intelligence-based screening for the detection of vision-threatening diabetic retinopathy. Random-effects meta-analysis forest plot of sensitivity for detecting vision-threatening diabetic retinopathy using artificial intelligence-based screening systems compared with manual reference grading. Sensitivity estimates are presented as logit-transformed event rates with 95 percent confidence intervals for individual studies and pooled effects. VTDR vision-threatening diabetic retinopathy, AI artificial intelligence, CI confidence interval.

The meta-analysis shown in Figs. 8 and 9 compares the pooled diagnostic sensitivity of SAF screening versus AI-based diagnostic systems for detecting VTDR, with sensitivity quantified as logit-transformed event rates and corresponding 95% confidence intervals. In Fig. 8, the SAF subgroup demonstrated generally high but variable sensitivity estimates across included screening implementations, with individual logit event rates ranging from 1.386 to 1.955, and study-level 95% confidence interval boundaries spanning from a lowest lower limit of 1.047 to a highest upper limit of 2.491, indicating that SAF workflows can detect VTDR with moderate-to-high sensitivity but with measurable dispersion across different imaging configurations and study settings. The pooled SAF sensitivity estimate was a logit event rate of 1.674, with a 95% confidence interval from 1.554 to 1.794, representing the overall sensitivity achieved by SAF approaches for identifying VTDR in the aggregated dataset. In contrast, Fig. 9 shows that the AI subgroup consistently achieved higher sensitivity estimates, with individual logit event rates ranging from 2.970 to 3.794, and study-level 95% confidence interval boundaries extending from a lowest lower limit of 1.925 to a highest upper limit of 5.372, reflecting uniformly strong VTDR detection performance across AI systems while also demonstrating that some studies exhibited wider confidence intervals and therefore reduced precision. The pooled AI sensitivity estimate was a logit event rate of 3.235, with a 95% confidence interval from 3.122 to 3.349, which is markedly higher than the pooled SAF estimate on the same logit scale, indicating a substantially greater probability of correctly identifying VTDR cases with AI-based screening. Because higher logit event rates correspond to higher sensitivity, back-transformation of the pooled values supports a clinically meaningful difference in true-positive detection between strategies, with the pooled SAF estimate corresponding to an approximate sensitivity of 84.2%, whereas the pooled AI estimate corresponds to an approximate sensitivity of 96.2%, demonstrating that AI-based diagnostic systems achieve substantially higher pooled sensitivity for VTDR detection and therefore a lower likelihood of missed sight-threatening disease compared with SAF screening within the evidence summarized by these forest plots.

### Meta-analysis: sensitivity for diabetic macular edema

**Fig. 10:**
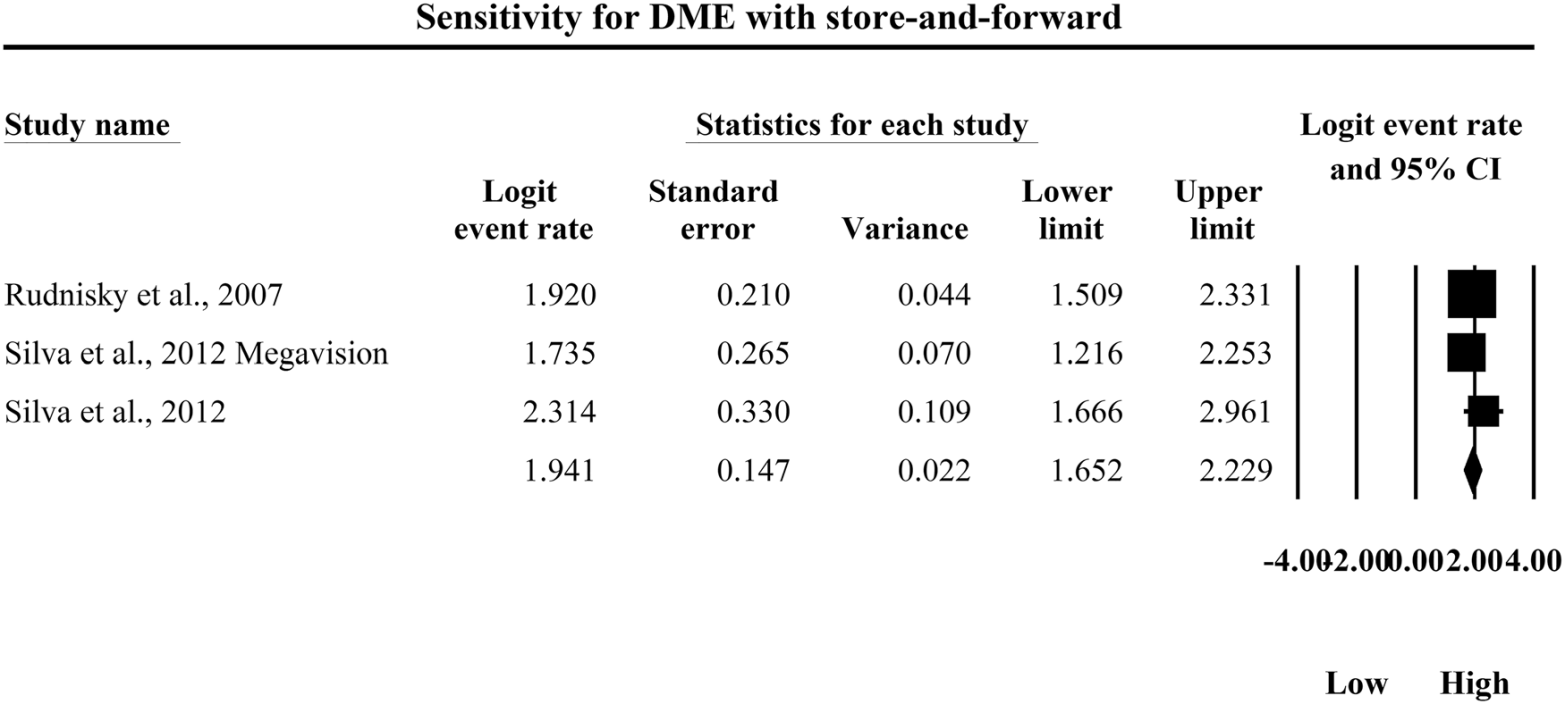
Diagnostic sensitivity of store-and-forward teleophthalmology for the detection of diabetic macular edema. Random-effects meta-analysis forest plot of sensitivity for detecting diabetic macular edema using store-and-forward teleophthalmology with human grading compared with manual reference grading. Sensitivity estimates are presented as logit-transformed event rates with 95 percent confidence intervals for individual studies and pooled effects. DME diabetic macular edema, SAF store-and-forward, CI confidence interval.

**Fig. 11:**
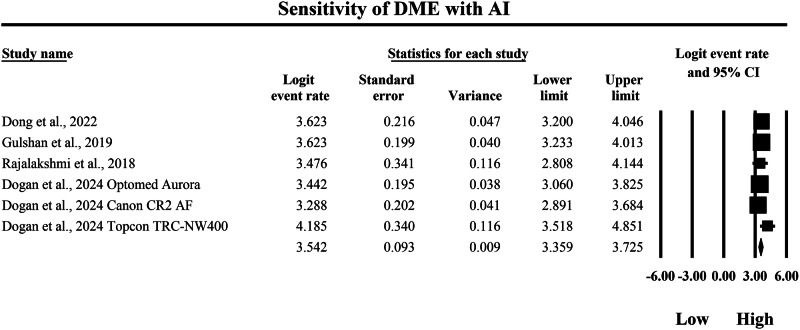
Diagnostic sensitivity of artificial intelligence-based screening for the detection of diabetic macular edema. Random-effects meta-analysis forest plot of sensitivity for detecting diabetic macular edema using artificial intelligence-based screening systems compared with manual reference grading. Sensitivity estimates are presented as logit-transformed event rates with 95 percent confidence intervals for individual studies and pooled effects. DME diabetic macular edema, AI artificial intelligence, CI confidence interval.

The meta-analysis presented in Figs. 10 and 11 compares the pooled diagnostic sensitivity of SAF screening versus AI-based diagnostic systems for detecting DME, with sensitivity estimates expressed as logit-transformed event rates and corresponding 95% confidence intervals. In Fig. 10, the SAF subgroup includes a limited number of study-level sensitivity estimates, with individual logit event rates ranging from 1.735 to 2.314, and study-specific 95% confidence interval boundaries extending from a lowest lower limit of 1.216 to a highest upper limit of 2.961, indicating that SAF workflows demonstrated moderately high sensitivity for DME detection but with measurable variability in precision across studies. The pooled SAF sensitivity estimate was a logit event rate of 1.941, with a 95% confidence interval from 1.652 to 2.229, representing the overall probability of correctly identifying true DME cases using SAF-based screening approaches in the aggregated evidence base. In contrast, Fig. 11 shows consistently higher sensitivity estimates in the AI subgroup, with individual logit event rates ranging from 3.288 to 4.185, and study-level 95% confidence interval boundaries spanning from a lowest lower limit of 2.891 to a highest upper limit of 4.851, demonstrating uniformly strong DME case detection performance across AI systems while also reflecting inter-study differences in precision as evidenced by varying confidence interval widths. The pooled AI sensitivity estimate was a logit event rate of 3.542, with a 95% confidence interval from 3.359 to 3.725, which is substantially higher than the pooled SAF estimate on the same logit scale and indicates a markedly greater probability of correctly identifying DME cases in the AI subgroup. Because higher logit event rates correspond to higher sensitivity, back-transformation of the pooled values supports a clinically meaningful difference in true-positive detection, with the pooled SAF estimate corresponding to an approximate sensitivity of 87.4%, whereas the pooled AI estimate corresponds to an approximate sensitivity of 97.2%, demonstrating that AI-based diagnostic systems achieve substantially higher pooled sensitivity for detecting DME and therefore a lower likelihood of missed DME cases compared with SAF screening within the set of studies summarized by these forest plots.

### Meta-analysis: specificity for any diabetic retinopathy

**Fig. 12:**
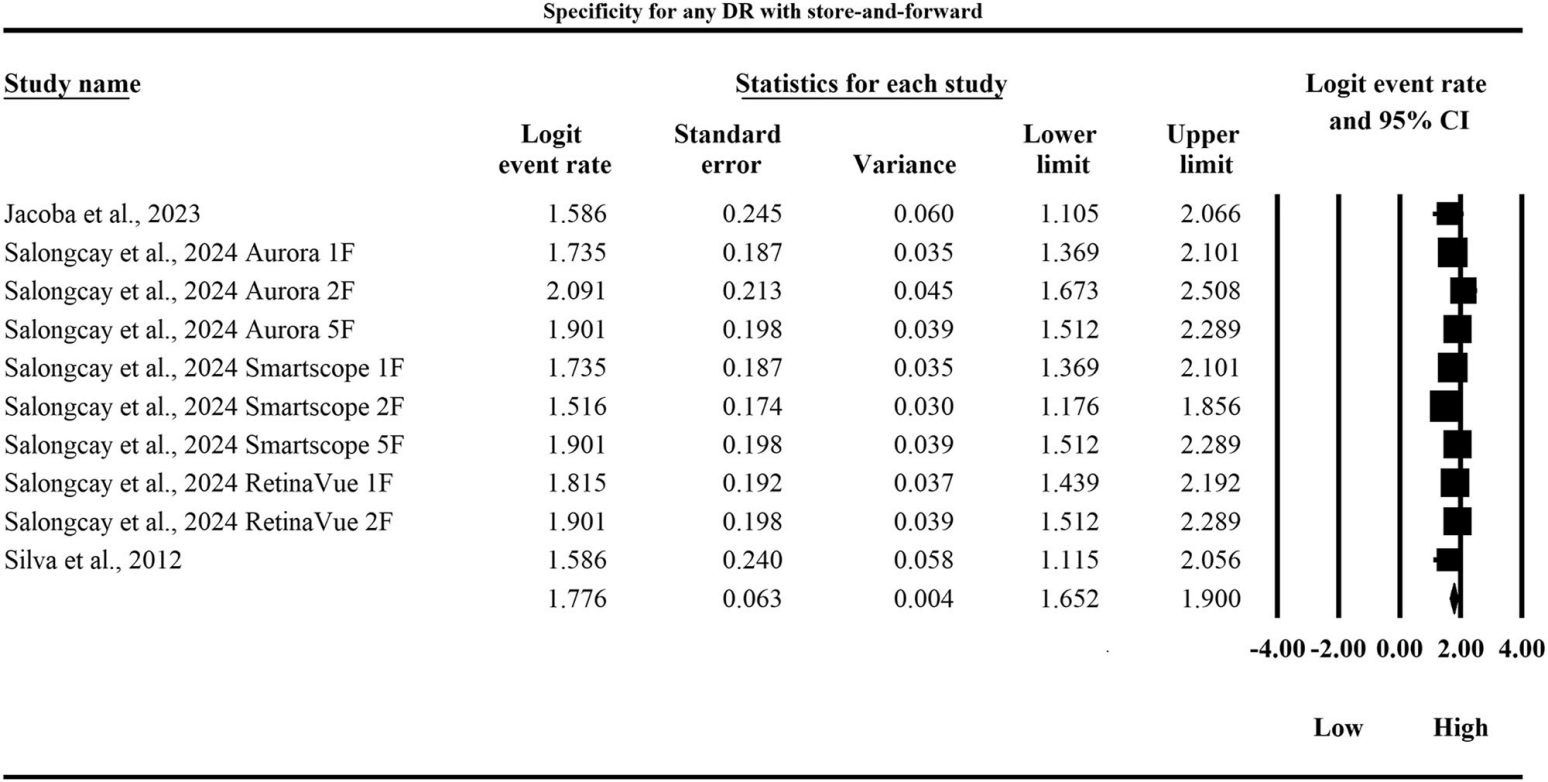
Diagnostic specificity of store-and-forward teleophthalmology for the exclusion of any diabetic retinopathy. Random-effects meta-analysis forest plot of specificity for excluding any diabetic retinopathy using store-and-forward teleophthalmology with human grading compared with manual reference grading. Specificity estimates are presented as logit-transformed event rates with 95 percent confidence intervals for individual studies and pooled effects. DR diabetic retinopathy, SAF store-and-forward, CI confidence interval.

**Fig. 13:**
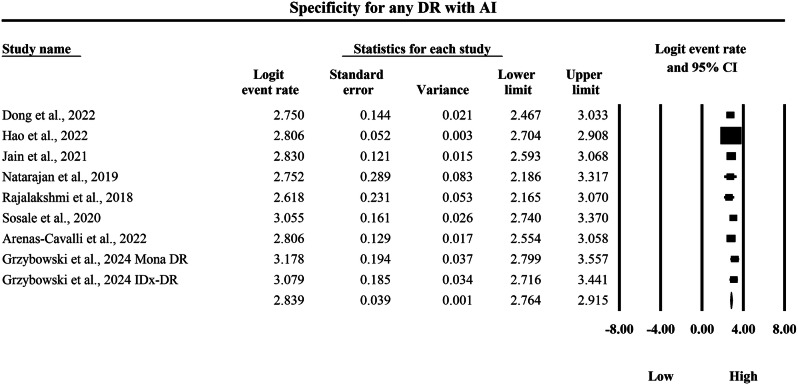
Diagnostic specificity of artificial intelligence-based screening for the exclusion of any diabetic retinopathy. Random-effects meta-analysis forest plot of specificity for excluding any diabetic retinopathy using artificial intelligence-based screening systems compared with manual reference grading. Specificity estimates are presented as logit-transformed event rates with 95 percent confidence intervals for individual studies and pooled effects. DR, diabetic retinopathy; AI artificial intelligence, CI confidence interval.

The meta-analysis presented in Figs. 12 and 13 provides a side-by-side descriptive comparison of the pooled diagnostic specificity for any DR between SAF and AI-based diagnostic systems, with specificity expressed as logit-transformed event rates and corresponding 95% CIs, where higher logit values indicate a greater probability of correctly classifying individuals without DR as test-negative. In Fig. 12, the SAF subgroup demonstrated study-specific logit specificity estimates ranging from 1.516 to 2.091, with individual 95% CI limits spanning from a lowest lower limit of 1.105 to a highest upper limit of 2.508, and the pooled SAF specificity was 1.776 with a 95% CI of 1.652 to 1.900, indicating a moderate-to-high overall ability of SAF screening to exclude non-DR cases. In Fig. 13, the AI subgroup showed consistently higher study-specific logit specificity estimates ranging from 2.618 to 3.178, with individual 95% CI limits spanning from a lowest lower limit of 2.165 to a highest upper limit of 3.557, and the pooled AI specificity was 2.839 with a 95% CI of 2.764 to 2.915, demonstrating a substantially stronger ability to correctly exclude individuals without DR compared with SAF. When back-transformed to the probability scale for clinical interpretability, the pooled logit specificity of 1.776 for SAF corresponds to an approximate specificity of 85.5%, whereas the pooled logit specificity of 2.839 for AI corresponds to an approximate specificity of 94.5%, indicating that AI-based systems achieve a markedly lower false-positive rate and would be expected to reduce unnecessary referrals relative to SAF screening for any DR. Overall, Figs. 12 and 13 provide clear evidence that, while both modalities demonstrate specificity values in a clinically useful range, AI yields a substantially higher pooled specificity with a relatively narrow pooled confidence interval, supporting a higher pooled specificity estimate within the AI evidence stream within screening populations.

### Meta-analysis: specificity for referable diabetic retinopathy

**Fig. 14:**
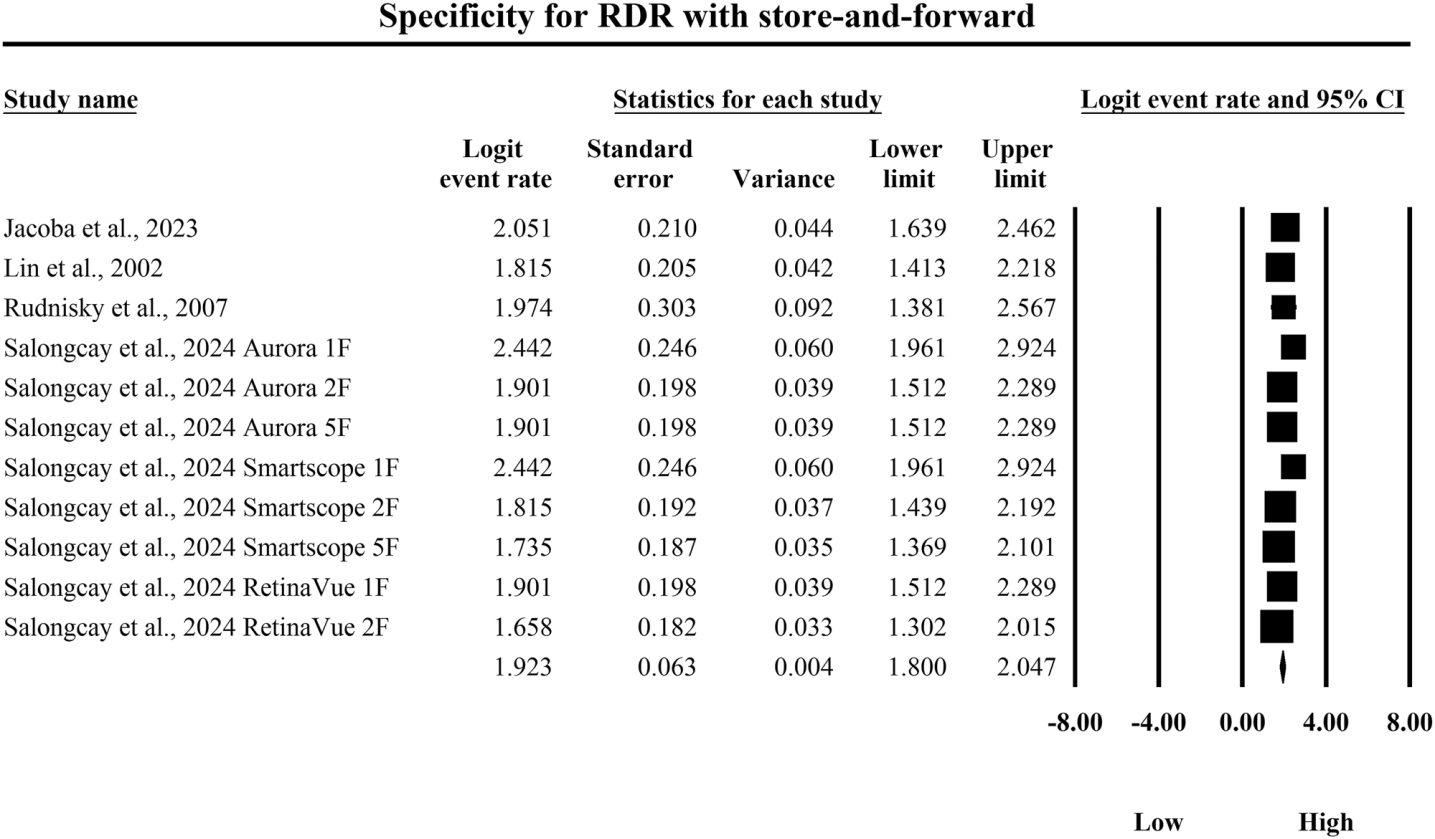
Diagnostic specificity of store-and-forward teleophthalmology for the exclusion of referable diabetic retinopathy. Random-effects meta-analysis forest plot of specificity for excluding referable diabetic retinopathy using store-and-forward teleophthalmology with human grading compared with manual reference grading. Specificity estimates are presented as logit-transformed event rates with 95 percent confidence intervals for individual studies and pooled effects. RDR referable diabetic retinopathy, SAF store-and-forward, CI confidence interval.

**Fig. 15:**
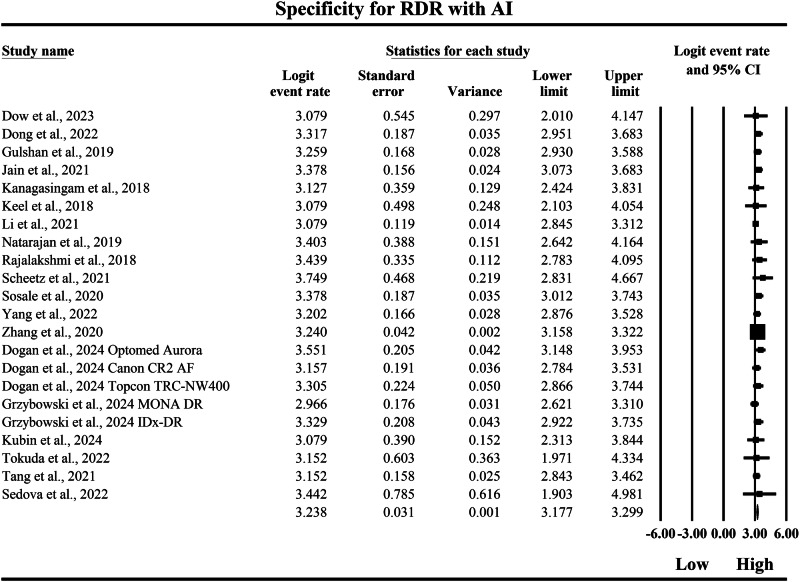
Diagnostic specificity of artificial intelligence-based screening for the exclusion of referable diabetic retinopathy. Random-effects meta-analysis forest plot of specificity for excluding referable diabetic retinopathy using artificial intelligence-based screening systems compared with manual reference grading. Specificity estimates are presented as logit-transformed event rates with 95 percent confidence intervals for individual studies and pooled effects. RDR referable diabetic retinopathy, AI artificial intelligence, CI confidence interval.

The meta-analysis presented in Figs. 14 and 15 compares the pooled diagnostic specificity of SAF screening versus AI-based diagnostic systems for identifying RDR, with specificity estimates expressed as logit-transformed event rates and corresponding 95% confidence intervals, where higher logit values indicate a greater probability of correctly classifying individuals without referable disease as negative. In Fig. 14, the SAF subgroup demonstrated moderately high specificity estimates with measurable variability across screening implementations, with individual logit event rates ranging from 1.658 to 2.442, and study-level 95% confidence interval boundaries extending from a lowest lower limit of 1.302 to a highest upper limit of 2.924, indicating that SAF workflows were generally effective in excluding non-referable cases but showed heterogeneity in precision across the included datasets. The pooled SAF specificity estimate was a logit event rate of 1.923, with a 95% confidence interval from 1.800 to 2.047, representing the overall specificity achieved by SAF-based screening approaches for RDR detection in the aggregated evidence base. In contrast, Fig. 15 shows consistently higher specificity estimates in the AI subgroup, with individual logit event rates ranging from 2.966 to 3.749, and study-level 95% confidence interval boundaries spanning from a lowest lower limit of 1.903 to a highest upper limit of 4.981, demonstrating that AI systems achieved a uniformly strong ability to exclude non-referable disease while also showing variability in confidence interval width across studies. The pooled AI specificity estimate was a logit event rate of 3.238, with a 95% confidence interval from 3.177 to 3.299, which is substantially higher than the pooled SAF estimate on the same logit scale and therefore indicates a markedly greater probability of correctly classifying non-RDR cases as negative using AI-based screening. Back-transformation of the pooled logit values further supports a clinically meaningful difference between strategies, with the pooled SAF estimate corresponding to an approximate specificity of 87.2%, whereas the pooled AI estimate corresponds to an approximate specificity of 96.2%, indicating that AI-based diagnostic systems achieve substantially higher pooled specificity for RDR and therefore a lower likelihood of false-positive classification and unnecessary referral compared with SAF screening within the set of studies summarized by these forest plots.

### Meta-analysis: specificity for vision-threatening diabetic retinopathy

**Fig. 16:**
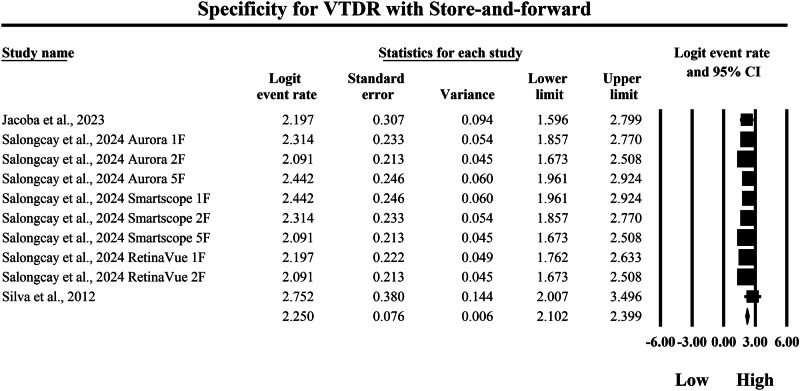
Diagnostic specificity of store-and-forward teleophthalmology for the exclusion of vision-threatening diabetic retinopathy. Random-effects meta-analysis forest plot of specificity for excluding vision-threatening diabetic retinopathy using store-and-forward teleophthalmology with human grading compared with manual reference grading. Specificity estimates are presented as logit-transformed event rates with 95 percent confidence intervals for individual studies and pooled effects. VTDR vision-threatening diabetic retinopathy, SAF store-and-forward, CI confidence interval.

**Fig. 17:**
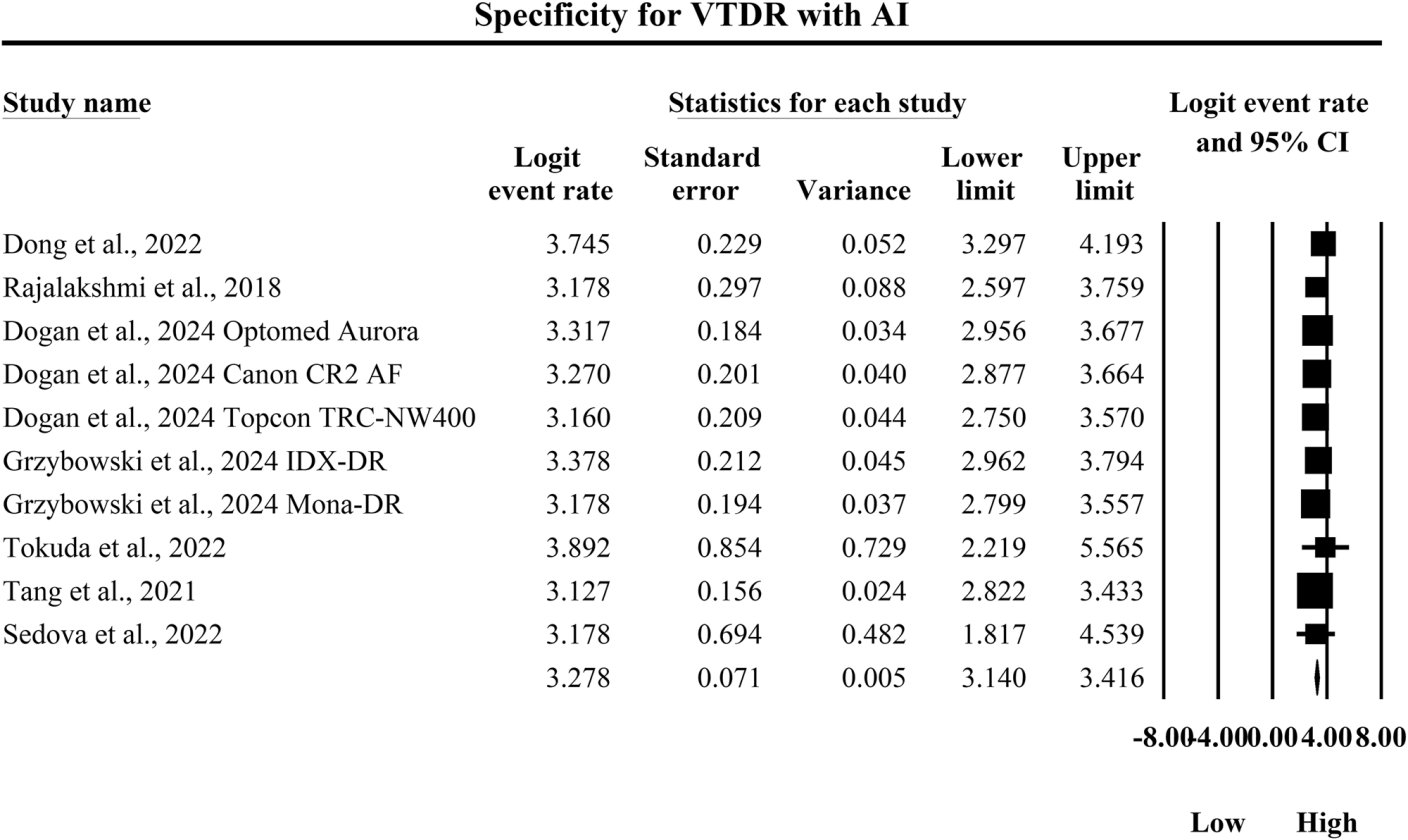
Diagnostic specificity of artificial intelligence-based screening for the exclusion of vision-threatening diabetic retinopathy. Random-effects meta-analysis forest plot of specificity for excluding vision-threatening diabetic retinopathy using artificial intelligence-based screening systems compared with manual reference grading. Specificity estimates are presented as logit-transformed event rates with 95 percent confidence intervals for individual studies and pooled effects. VTDR vision-threatening diabetic retinopathy, AI artificial intelligence, CI confidence interval.

The meta-analysis presented in Figs. 16 and 17 compares the pooled diagnostic specificity of SAF screening versus AI-based diagnostic systems for identifying VTDR, with specificity quantified as logit-transformed event rates and corresponding 95% confidence intervals, where higher logit values indicate a greater probability of correctly classifying individuals without VTDR as negative. In Fig. 16, the SAF subgroup demonstrated generally high specificity estimates across included screening implementations, with individual logit event rates ranging from 2.091 to 2.752, and study-level 95% confidence interval boundaries extending from a lowest lower limit of 1.596 to a highest upper limit of 3.496, indicating that SAF workflows were consistently capable of excluding non-VTDR cases but with moderate variability in estimate precision across studies. The pooled SAF specificity estimate was a logit event rate of 2.250, with a 95% confidence interval from 2.102 to 2.399, representing the overall specificity achieved by SAF-based screening approaches for VTDR in the aggregated evidence base. In contrast, Fig. 17 shows higher specificity estimates in the AI subgroup, with individual logit event rates ranging from 3.127 to 3.892, and study-level 95% confidence interval boundaries spanning from a lowest lower limit of 1.817 to a highest upper limit of 5.565, demonstrating strong ability of AI systems to correctly exclude non-VTDR cases while also showing that some studies exhibited wider confidence intervals and therefore reduced precision. The pooled AI specificity estimate was a logit event rate of 3.278, with a 95% confidence interval from 3.140 to 3.416, which is substantially higher than the pooled SAF estimate on the same logit scale and therefore indicates a markedly greater probability of correctly classifying individuals without VTDR using AI-based screening. Back-transformation of the pooled logit values supports a clinically meaningful difference in specificity between strategies, with the pooled SAF estimate corresponding to an approximate specificity of 90.5%, whereas the pooled AI estimate corresponds to an approximate specificity of 96.4%, indicating that AI-based diagnostic systems achieve substantially higher pooled specificity for VTDR and therefore a lower likelihood of false-positive VTDR classification and unnecessary urgent referral compared with SAF screening within the studies summarized by these forest plots.

### Meta-analysis: specificity for diabetic macular edema

**Fig. 18:**
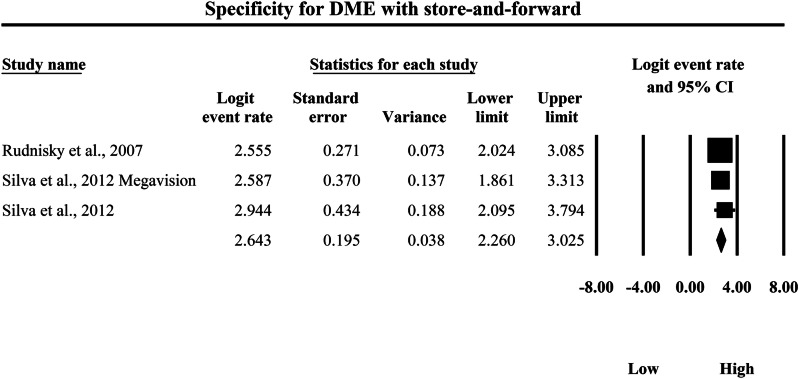
Diagnostic specificity of store-and-forward teleophthalmology for the exclusion of diabetic macular edema. Random-effects meta-analysis forest plot of specificity for excluding diabetic macular edema using store-and-forward teleophthalmology with human grading compared with manual reference grading. Specificity estimates are presented as logit-transformed event rates with 95 percent confidence intervals for individual studies and pooled effects. DME diabetic macular edema, SAF store-and-forward, CI confidence interval.

**Fig. 19:**
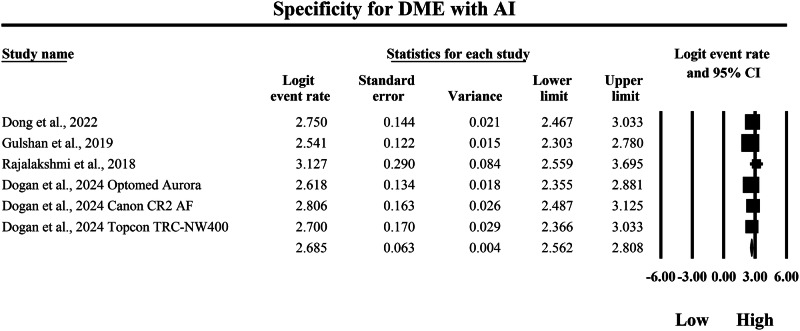
Diagnostic specificity of artificial intelligence-based screening for the exclusion of diabetic macular edema. Random-effects meta-analysis forest plot of specificity for excluding diabetic macular edema using artificial intelligence-based screening systems compared with manual reference grading. Specificity estimates are presented as logit-transformed event rates with 95 percent confidence intervals for individual studies and pooled effects. DME diabetic macular edema, AI artificial intelligence, CI confidence interval.

The meta-analysis presented in Figs. 18 and 19 compares the pooled diagnostic specificity of SAF screening versus AI-based diagnostic systems for detecting DME, with specificity quantified as logit-transformed event rates and corresponding 95% confidence intervals, where higher logit values indicate a greater probability of correctly classifying individuals without DME as negative. In Fig. 18, the SAF subgroup demonstrated consistently high specificity estimates across the included studies, with individual logit event rates ranging from 2.555 to 2.944, and study-level 95% confidence interval boundaries extending from a lowest lower limit of 1.861 to a highest upper limit of 3.794, indicating that SAF workflows generally performed well in excluding non-DME cases while also showing variability in estimate precision across studies. The pooled SAF specificity estimate was a logit event rate of 2.643, with a 95% confidence interval from 2.260 to 3.025, representing the overall specificity achieved by SAF-based screening approaches for DME detection within the aggregated evidence base. In Fig. 19, the AI subgroup likewise demonstrated high specificity estimates, with individual logit event rates ranging from 2.541 to 3.127, and study-level 95% confidence interval boundaries spanning from a lowest lower limit of 2.303 to a highest upper limit of 3.695, demonstrating strong and relatively consistent ability of AI systems to correctly exclude individuals without DME across the included datasets. The pooled AI specificity estimate was a logit event rate of 2.685, with a 95% confidence interval from 2.562 to 2.808, which is very close to the pooled SAF estimate on the same logit scale and indicates broadly comparable overall specificity between AI-based and SAF screening modalities for DME detection in this dataset. Back-transformation of the pooled logit values further supports this similarity, with the pooled SAF estimate corresponding to an approximate specificity of 93.4%, and the pooled AI estimate corresponding to an approximate specificity of 93.6%, indicating that both approaches achieve a similarly high probability of correctly excluding non-DME cases and therefore generate comparably low false-positive rates for DME classification within the studies summarized by these forest plots.

### Meta-regression: AI sensitivity-specificity relationship

**Fig. 20:**
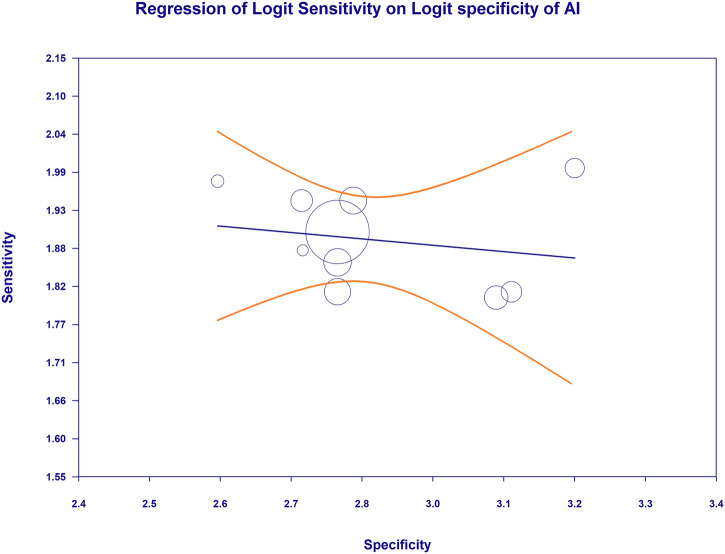
Meta regression of sensitivity and specificity for artificial intelligence in the detection of any diabetic retinopathy. Bubble plot meta-regression assessing the study-level association between logit sensitivity and logit specificity for artificial intelligence-based screening systems for the detection of any diabetic retinopathy. Circle size is proportional to study weight. The fitted regression line indicates the predicted relationship, with confidence bands reflecting uncertainty. DR diabetic retinopathy, AI artificial intelligence.

The meta-regression analysis shown in Fig. 20 evaluates the study-level relationship between AI diagnostic sensitivity and AI diagnostic specificity for detecting any DR, with both parameters expressed on the logit scale, as indicated by the regression of logit sensitivity on logit specificity. The bubble plot demonstrates that study estimates cluster within a relatively narrow operating range, with logit specificity values concentrated around approximately 2.6–3.2 and logit sensitivity values concentrated around approximately 1.8–2.0, while the fitted regression line is essentially flat with a slight negative slope, indicating that increasing logit specificity across studies is not associated with any meaningful increase or decrease in logit sensitivity. The distribution of bubbles, including the presence of a large, high-weight bubble near the center of the plot and smaller bubbles dispersed around it, further suggests that the overall trend is not driven by any single influential study and that the observed sensitivity remains broadly stable across the range of specificities represented. In addition, the wide curved confidence bands around the fitted regression line indicate substantial uncertainty around the predicted sensitivity at the extremes of specificity, while the dense clustering of points near the middle of the specificity range supports a consistent central tendency in AI performance for detecting any DR. Collectively, Fig. 20 provides visual evidence that across the included studies, AI-based screening systems do not demonstrate a strong study-level sensitivity-specificity trade-off for any DR detection within the specificity range evaluated, supporting the interpretation that differences in specificity across studies are not accompanied by systematic or clinically meaningful shifts in sensitivity on the logit scale.

### Meta-regression: store-and-forward sensitivity-specificity relationship

**Fig. 21:**
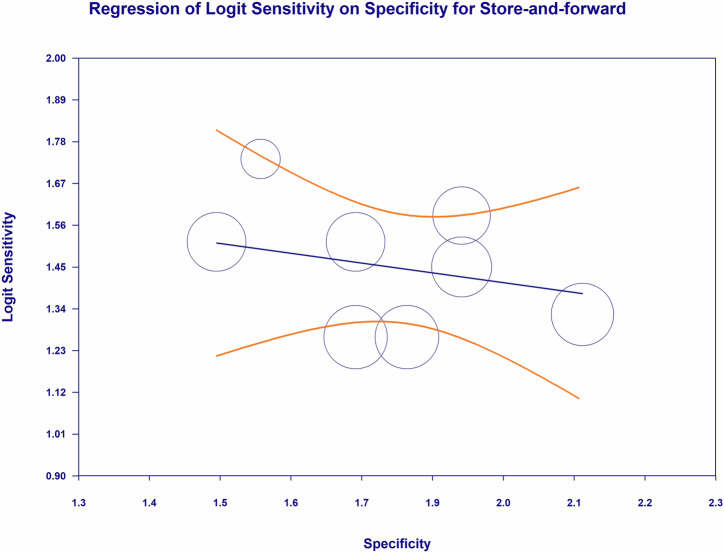
Meta regression of sensitivity and specificity for store-and-forward teleophthalmology in the detection of any diabetic retinopathy. Bubble plot meta-regression assessing the study-level association between logit sensitivity and logit specificity for store-and-forward teleophthalmology for the detection of any diabetic retinopathy. Circle size is proportional to study weight. The fitted regression line indicates the predicted relationship, with confidence bands reflecting uncertainty. DR diabetic retinopathy, SAF store-and-forward.

The meta-regression analysis shown in Fig. 21 evaluates the study-level relationship between SAF diagnostic sensitivity and specificity for detecting any DR, with logit sensitivity plotted on the y-axis and specificity plotted on the x-axis, thereby assessing whether differences in specificity across SAF screening implementations are associated with systematic variation in sensitivity. Visual inspection of the bubble plot demonstrates that study estimates cluster within a relatively narrow operating range, with specificity values concentrated approximately between 1.5 and 2.1 and logit sensitivity values concentrated approximately between 1.2 and 1.7, while the fitted regression line shows a shallow downward slope, indicating that increases in specificity across studies are not accompanied by any clear or clinically meaningful increase in sensitivity and, if anything, correspond to only a minimal decline in sensitivity on the logit scale. The distribution of bubbles, including several larger bubbles in the central region of the plot and smaller bubbles dispersed across the observed specificity range, suggests that the overall trend is not dominated by a single influential estimate and that SAF sensitivity remains broadly stable across the range of specificities evaluated. The wide, curved confidence bands surrounding the fitted regression line further indicate substantial uncertainty around the predicted sensitivity at the extremes of specificity, reinforcing the absence of a strong or consistent sensitivity-specificity trade-off within the body of evidence summarized. Overall, Fig. 21 provides visual evidence that, for SAF teleophthalmology applied to the detection of any DR, between-study variation in specificity is not associated with a meaningful systematic shift in sensitivity, supporting the interpretation that SAF screening performance for any DR detection is relatively stable across different implementations within the specificity range represented in these included study estimates.

### Publication bias assessment

Across all eight outcomes (Supplementary Figs. 1–16), publication bias was minimal to absent for sensitivity outcomes and most specificity outcomes, with only mild small-study effects detected for RDR and VTDR specificity. For the sensitivity of Any DR, publication bias assessment demonstrated no meaningful small-study effects. Rosenthal’s fail-safe *N* was >10,000, indicating that an extremely large number of unpublished null studies would be required to negate the pooled effect. Egger’s regression intercept was non-significant (*p* > 0.05), and Begg–Mazumdar rank correlation was also non-significant, supporting funnel plot symmetry. This aligns with forest plots showing tight clustering of study estimates and narrow confidence intervals, dominated by large validation studies, suggesting robust and unbiased pooled sensitivity estimates. For the specificity of Any DR, fail-safe *N* values exceeded 9000, supporting high result stability. Egger’s test was non-significant (*p* > 0.05), while Begg’s test showed no significant rank correlation, indicating minimal publication bias. Forest plots demonstrated limited dispersion and balanced study weights, with large AI validation cohorts contributing most of the weight. Trim-and-fill analysis did not impute additional studies, and pooled specificity remained unchanged. Sensitivity analyses for RDR revealed very large fail-safe *N* values (>20,000), confirming robustness. Egger’s intercept was non-significant, and Begg’s test did not indicate asymmetry. Corresponding forest plots showed highly consistent sensitivity estimates, narrow confidence intervals, and dominance of large multicenter studies. No imputed studies were added under trim-and-fill, confirming minimal publication bias. For RDR specificity, fail-safe N values remained substantial (>5000), but Egger’s regression intercept reached borderline statistical significance (*p* < 0.05), and Begg’s test demonstrated a significant rank correlation, suggesting potential small-study effects. Forest plots showed greater spread of effect sizes and wider confidence intervals among smaller studies, consistent with mild asymmetry. However, trim-and-fill adjustment resulted in only marginal attenuation (<2–3%) of pooled specificity, without altering clinical interpretation. For VTDR sensitivity, Rosenthal’s fail-safe *N* exceeded 30,000, indicating exceptional robustness. Both Egger’s test and Begg’s test were non-significant, supporting the absence of publication bias. Forest plots showed tight clustering around the pooled estimate, with large studies contributing the majority of statistical weight. Trim-and-fill did not impute missing studies, reinforcing result stability. Specificity analyses for VTDR showed moderate fail-safe *N* values (>3000). Egger’s intercept was borderline significant, while Begg’s test indicated weak but statistically detectable rank correlation, suggesting limited publication bias. Forest plots revealed moderate dispersion, particularly among smaller studies, though pooled estimates remained stable. Trim-and-fill correction produced minimal effect size reduction, confirming that bias did not materially affect conclusions. For DME sensitivity, fail-safe *N* values were >8000, and both Egger’s and Begg’s tests were non-significant, indicating no evidence of publication bias. Forest plots demonstrated consistent effect sizes with narrow confidence intervals, despite the smaller number of included studies. No studies were imputed under trim-and-fill, supporting unbiased pooled sensitivity estimates. Specificity for DME showed low heterogeneity and fail-safe *N* values > 2000. Egger’s regression intercept and Begg–Mazumdar test were both non-significant, and trim-and-fill analysis did not adjust the pooled estimate. Forest plots exhibited symmetrical distribution and limited CI overlap, indicating stable and unbiased specificity estimates across both AI and store-and-forward studies.

### Clinical utility translation: expected referrals and missed cases across settings

To translate pooled diagnostic operating characteristics into clinically interpretable implementation outcomes, pooled sensitivity and specificity estimates were converted into expected screening consequences per 1000 individuals screened across three illustrative prevalence scenarios (5%, 15%, and 30%), representing low-prevalence population screening, moderate-risk clinical screening, and high-risk referral-oriented settings, respectively. These analyses were performed for the key screening endpoints of any diabetic retinopathy (DR), referable DR (RDR), and vision-threatening diabetic retinopathy (VTDR), with particular emphasis on VTDR because missed detection at this stage carries the greatest risk of preventable vision loss.

For any DR at a prevalence of 5%, pooled operating characteristics suggest that AI-based pathways would identify approximately 43 true-positive cases and miss about 7 cases per 1000 screened, while generating fewer false-positive referrals compared with SAF workflows due to higher pooled specificity. Under the same scenario, SAF screening would detect fewer true-positive cases and produce a larger number of false-positive referrals, reflecting the trade-off between sensitivity and specificity observed across pathway-specific evidence streams. As prevalence increased to 15% and 30%, absolute differences in detected cases and referral burden widened, demonstrating how identical operating characteristics translate into markedly different real-world consequences depending on baseline disease risk.

For referable DR, decision consequences were more clinically meaningful because referral allocation becomes directly relevant to service capacity. At a moderate prevalence of 15%, AI-based screening would be expected to detect approximately 144 true RDR cases per 1000 screened, with fewer missed cases compared with SAF, while maintaining a manageable referral burden due to high pooled specificity. SAF workflows, while producing slightly fewer unnecessary referrals in certain scenarios, showed a higher number of missed referral-relevant cases, reflecting lower pooled sensitivity for this endpoint. At higher prevalence settings (30%), both pathways generated increased referral volumes; however, the absolute number of missed RDR cases remained lower in the AI pathway, consistent with its higher sensitivity operating profile.

For VTDR, the endpoint is most closely linked to the immediate risk of irreversible vision loss; the clinical utility translation demonstrated the clearest divergence between pathways. At a prevalence of 5%, AI-based screening would be expected to miss only a small number of VTDR cases per 1000 screened, whereas SAF pathways would miss a larger absolute number despite maintaining high specificity. At moderate prevalence (15%), AI-based screening was associated with substantially fewer missed-VTDR cases and higher true-positive detection, while SAF workflows generated slightly fewer false-positive urgent referrals. In high-prevalence settings (30%), both pathways produced increased referral volumes; however, AI-based screening maintained a lower missed-VTDR burden, emphasizing its suitability when minimizing false negatives is prioritized.

Across all prevalence scenarios, total referral volume reflected the combined contribution of true-positive and false-positive classifications. AI pathways generally produced higher true-positive yield with acceptable referral burden, whereas SAF pathways demonstrated stable performance with relatively strong rule-out characteristics. These findings illustrate that clinical utility is inherently setting-dependent and cannot be inferred from sensitivity or specificity alone.

From an implementation perspective, these decision-consequence analyses support complementary strategic roles for the two screening pathways. In environments where screening capacity or specialist availability constrains referral volume, SAF workflows may be preferentially positioned due to their stable specificity and predictable referral load. Conversely, in high-throughput screening programs prioritizing maximal detection of referral-relevant disease, particularly for VDTR, AI-based pathways may provide greater clinical utility by reducing missed cases while maintaining clinically acceptable referral burden. Collectively, these results reinforce that AI and SAF should be interpreted as pathway-specific operating strategies rather than competing tests, and that optimal deployment depends on local prevalence, referral capacity, and decision priorities (Supplementary Table 1).

## Discussion

This PRISMA-DTA-aligned systematic review and meta-analysis provides a comprehensive, implementation-focused synthesis of diagnostic performance evidence for two major population-level diabetic retinopathy (DR) screening paradigms: autonomous artificial intelligence (AI)-based screening systems and store-and-forward (SAF) teleophthalmology interpreted by human graders. This work is particularly relevant for the United States, where diabetes prevalence is substantial, and DR screening implementation remains heterogeneous across healthcare settings, creating an urgent need for scalable models that can be integrated into primary care and community workflows to reduce preventable vision loss at the population scale. To our knowledge, this is among the first PRISMA-DTA-aligned evidence syntheses to explicitly distinguish AI-based screening and SAF teleophthalmology as two fundamentally different teleophthalmology pathways and to quantitatively characterize their operating characteristics across clinically meaningful disease-severity thresholds, including any DR, referable DR (RDR), vision-threatening DR (VTDR), and diabetic macular edema (DME), within a unified analytic framework.

Across pooled analyses, AI-based systems demonstrated consistently high sensitivity for referral-relevant endpoints, particularly RDR, VTDR, and DME, supporting their role as scalable triage tools in high-throughput screening environments. SAF teleophthalmology also demonstrated robust diagnostic capability, reflecting the enduring clinical utility of structured remote human interpretation when standardized imaging and grading protocols are applied. Because AI and SAF evidence streams represent distinct screening ecosystems differing in patient case-mix, imaging acquisition protocols, DR prevalence, reference grading workflows, and operational thresholds, our modality-stratified meta-analytic approach was intentionally designed to generate pathway-specific performance profiles rather than direct comparative claims^15^. These pathway-level performance estimates provide actionable evidence for referral planning, clinical pathway design, and future trial development.

Specificity findings further indicate that both pathways can achieve clinically meaningful rule-out performance, thereby limiting unnecessary referrals and supporting screening sustainability. Referral efficiency is critical in large health systems, where excessive false-positive volume may overwhelm retina services and delay care for truly referable disease. Notably, DME specificity was broadly comparable between modalities, suggesting that color fundus photography-based DME ascertainment and shared reference-standard constraints may impose similar diagnostic ceilings regardless of whether classification is performed by AI or SAF graders. These findings reinforce that screening performance should be interpreted in the context of pathway design rather than isolated accuracy metrics.

Our pooled sensitivity estimates are consistent with pivotal validation studies of FDA-cleared autonomous AI systems. For example, IDx-DR demonstrated sensitivity of 96.8% for more-than-mild DR in a multisite U.S. study^48^, while EyeArt achieved 95.8% sensitivity for referable DR in a UK screening population^49^. These data support the clinical plausibility and translational relevance of the pooled estimates observed in this analysis, particularly as autonomous AI systems are increasingly deployed in U.S. primary care and endocrinology settings. Importantly, our findings do not imply an unavoidable specificity penalty for AI; rather, they suggest that high-sensitivity operating points can coexist with clinically acceptable specificity depending on threshold calibration and integration within screening workflows^50^. Inter-study variability remains expected because imaging protocols, case-mix severity, ungradable image handling, and adjudication processes may influence apparent operating characteristics even among similar algorithmic systems^51^.

Similarly, the pooled specificity observed for SAF workflows aligns with evidence demonstrating that trained human graders in structured telemedicine programs effectively exclude non-referable disease^52^. Reported sensitivities in established programs such as the English National Screening Programme (87–94%) are consistent with our pooled estimates^53^. Moderate heterogeneity in SAF performance likely reflects differences in grader expertise and adjudication procedures, factors previously shown to influence diagnostic consistency in teleophthalmology programs^54^.

A central implication of this study is that screening strategy selection should be interpreted as a real-world clinical utility problem, rather than purely a diagnostic accuracy comparison. Referral thresholds are operational policy decisions shaped by local prevalence, specialist capacity, and tolerance for misclassification. The trade-off between sensitivity and specificity represents a fundamental principle of diagnostic-test performance^55^; however, the clinical impact depends on how false negatives and false positives are weighted in practice. In settings where preventing missed RDR or VTDR is prioritized, such as large U.S. screening initiatives targeting vision-loss prevention and health-equity improvement, AI-based pathways may function effectively as front-line triage systems^56^. Conversely, where specialist capacity is constrained and minimizing unnecessary referrals is critical, SAF workflows supported by structured human grading may remain highly effective^57^. Hybrid deployment models, in which AI performs initial screening followed by human over-read of uncertain cases, may optimize both efficiency and safety^9^.

To directly address clinical utility, pooled operating characteristics were interpreted through decision thresholds, misclassification costs, and net-benefit concepts. Decision-curve methodology formalizes this framework by weighting false positives relative to true positives using threshold probability (p_t_), thereby translating diagnostic performance into outcome-oriented decision value. Using pooled RDR operating points at a prevalence of 15%, AI yielded an estimated net benefit of 140.7 net true-positive decisions per 1000 screened compared with 120.8 per 1000 for SAF at p_t_ = 0.10, both outperforming “refer-all” (55.6) and “refer-none” strategies. At a stricter threshold (p_t_ = 0.20), AI retained higher net benefit (136.2 per 1000) versus SAF (105.7 per 1000), while indiscriminate referral became harmful (−62.5 per 1000). These findings illustrate how pathway preference depends on decision thresholds and misclassification weighting rather than raw accuracy metrics alone, emphasizing that clinical utility emerges from consequence-based evaluation.

Compared with recent systematic reviews by Alqahtani et al.^58^ and Tahir et al.^59^, the present study introduces several methodological and conceptual advances. Prior reviews largely treated manual grading as a homogeneous comparator, whereas our study explicitly distinguishes autonomous AI and SAF teleophthalmology as separate pathway-level evidence streams. Unlike analyses pooling mixed designs without addressing pathway heterogeneity, this study applied diagnostic-test-accuracy-specific methodology, including logit transformation, random-effects modeling, meta-regression, and structured handling of multi-algorithm datasets. Furthermore, our analysis integrates real-world workflow considerations-including imaging acquisition, population context, referral thresholds, and implementation constraints-providing implementation-relevant performance ranges rather than superiority claims. By reframing AI and SAF as parallel but non-comparable evidence streams, this work avoids invalid indirect comparisons and establishes a modality-stratified benchmark to guide future pathway-controlled prospective trials.

Key strengths include separate synthesis of sensitivity and specificity using logit transformation and random-effects pooling, clinically meaningful severity-threshold stratification (any DR, RDR, VTDR, DME), structured handling of correlated multi-algorithm datasets, and an implementation-oriented framework directly applicable to program design and guideline development. These features support the long-term translational relevance of this study for both U.S. and global screening implementation.

Several limitations should be acknowledged. First, AI and SAF evidence streams were derived from largely non-overlapping populations and workflows, precluding strict head-to-head inference; however, the pathway-stratified approach reflects the most policy-relevant evidence currently available. Second, variability in imaging protocols, ungradable image handling, and reference grading procedures may contribute to heterogeneity despite the use of robust statistical approaches. Third, the SAF evidence base was smaller than the AI literature, which may limit precision for some subgroup estimates; nevertheless, pooled SAF performance remained stable and clinically interpretable. Finally, while decision-consequence analyses provide clinically meaningful approximations of implementation impact, individual-level risk modeling was not feasible, and future studies incorporating full decision-curve analyses would further strengthen clinical utility evaluation.

Future research should prioritize prospective within-cohort comparisons of AI-first, SAF-first, and hybrid workflows using standardized imaging and reference grading protocols^46^. Beyond diagnostic accuracy, studies evaluating cost-effectiveness, workflow integration, long-term patient outcomes, and equity of access are essential to inform policy decisions, particularly in resource-limited settings^60^. Standardized reporting of AI operating thresholds and reference-standard definitions will further improve evidence synthesis and comparability across studies^61^.

Beyond diagnostic performance, successful deployment of AI-enabled screening systems requires robust governance frameworks addressing transparency, continuous monitoring, algorithm drift, equity, accountability, and appropriate human oversight. Recent consensus initiatives, including the FUTURE-AI framework, emphasize that clinical AI systems should be evaluated not only for diagnostic accuracy but also for safety, fairness, explainability, and lifecycle governance spanning development, validation, deployment, and post-implementation monitoring. These principles are particularly relevant in population-level screening programs, where algorithm performance may evolve over time due to shifts in imaging devices, disease prevalence, or patient demographics. Incorporating governance considerations reinforces the interpretation of AI and SAF screening as implementation strategies embedded within healthcare ecosystems rather than isolated diagnostic tools. From a policy perspective, governance-aware deployment may improve trust, facilitate regulatory alignment, and reduce risks associated with unintended bias or performance degradation, thereby enhancing the long-term sustainability of digital screening pathways.

In conclusion, this PRISMA-DTA-compliant synthesis provides a modality-stratified benchmark for population-based DR screening pathways and demonstrates that AI-based and SAF teleophthalmology represent complementary implementation strategies rather than directly competing technologies. Quantitatively, AI pathways showed high pooled sensitivity for referral-relevant endpoints including RDR, VTDR, and DME, whereas SAF workflows retained clinically useful specificity and stable real-world rule-out performance. Decision-analytic translation showed that, at a prevalence of 15%, AI produced higher net benefit (140.7 vs 120.8 net true-positive decisions per 1000 screened at p_t_ = 0.10), illustrating the impact of decision thresholds and misclassification costs on pathway selection. These findings emphasize that optimal deployment depends on local prevalence, referral capacity, and acceptable trade-offs between missed disease and referral burden. By integrating diagnostic accuracy with decision-analytic clinical utility and outcome-focused interpretation, this study provides an implementation-ready evidence foundation to guide next-generation DR screening strategies aimed at reducing preventable vision loss.

## Methods

This systematic review and meta-analysis were conducted following Preferred Reporting Items for Systematic Reviews and Meta-Analyses of Diagnostic Test Accuracy studies (PRISMA-DTA) guidelines^62^ and were governed by a prospectively registered protocol that pre-specified eligibility criteria, search methods, data extraction procedures, risk-of-bias assessments, and statistical analysis plans. Our systematic review has been registered on an online registration website, PROSPERO, with the number CRD42024557212.

### Ethics approval and informed consent

This systematic review and meta-analysis used data extracted from previously published studies and did not involve new enrolment of human participants, access to identifiable private information, or collection of biological specimens. Therefore, ethics committee approval and informed consent were not required for this study, and a formal ethics approval waiver was not applicable. As no institutional ethics review was sought or required, no approving ethics committee name or application reference number is available for this study.

### Information sources and search strategy

A systematic literature search was conducted to identify studies evaluating the diagnostic accuracy of artificial intelligence (AI)-based systems and store-and-forward teleophthalmology for diabetic retinopathy screening. Six electronic databases (PubMed/MEDLINE, Embase, Web of Science, Scopus, the Cochrane Library, and Google Scholar) were searched from inception until December 29th, 2025. The search strategy (Table 2) combined controlled vocabulary terms and free-text keywords related to diabetic retinopathy, diabetic macular edema, referable and vision-threatening diabetic retinopathy, artificial intelligence, deep learning, teleophthalmology, store-and-forward screening, fundus photography, sensitivity, and specificity. Boolean operators and database-specific syntax were applied to optimize retrieval. The search was independently conducted by two authors to ensure completeness and reduce selection bias. All retrieved records were imported into reference management software, and duplicates were removed prior to screening. Titles and abstracts were screened independently, followed by full-text assessment of eligible studies. Discrepancies were resolved by consensus. Reference lists of included studies and relevant reviews were manually searched to identify additional eligible articles.

**Table 2:** Search strategy employed

Database	Search string
PubMed / MEDLINE	(“Diabetic Retinopathy” OR “diabetic macular edema” OR DR OR DME OR RDR OR VTDR) AND (“Artificial Intelligence” OR AI OR “Deep Learning” OR “Machine Learning” OR “Neural Network”) AND (“teleophthalmology” OR “store and forward” OR telemedicine OR “remote screening”) AND (“sensitivity” OR “specificity” OR “diagnostic accuracy”)
Embase	(‘diabetic retinopathy’/exp OR ‘diabetic macular edema’ OR DR OR DME) AND (‘artificial intelligence’/exp OR ‘deep learning’ OR ‘machine learning’) AND (‘teleophthalmology’ OR ‘store and forward’ OR telemedicine) AND (‘diagnostic accuracy’ OR sensitivity OR specificity)
Web of Science	TS = ((“diabetic retinopathy” OR “diabetic macular edema” OR DR OR DME OR RDR OR VTDR) AND (“artificial intelligence” OR “deep learning” OR “machine learning”) AND (“teleophthalmology” OR “store and forward” OR telemedicine) AND (“diagnostic accuracy” OR sensitivity OR specificity))
Scopus	TITLE-ABS-KEY (“diabetic retinopathy” OR “diabetic macular edema” OR DR OR DME OR RDR OR VTDR) AND TITLE-ABS-KEY (“artificial intelligence” OR “deep learning” OR “machine learning”) AND TITLE-ABS-KEY (“teleophthalmology” OR “store and forward” OR telemedicine) AND TITLE-ABS-KEY (sensitivity OR specificity OR “diagnostic accuracy”)
Cochrane Library	(“diabetic retinopathy” OR “diabetic macular edema”) AND (“artificial intelligence” OR “deep learning”) AND (“teleophthalmology” OR “store and forward”) AND (“diagnostic accuracy” OR sensitivity OR specificity)
Google Scholar	“diabetic retinopathy” AND (“artificial intelligence” OR “deep learning”) AND (“store and forward” OR teleophthalmology) AND (sensitivity OR specificity OR diagnostic accuracy)

### Study selection and eligibility criteria

Two research authors (K.-Y.C. and H.-C.C.) independently screened the titles and abstracts of all unique records against the established eligibility criteria. Any disagreements were settled by discussion or the intervention of a third author (C.-M.C.) to find a consensus. The remaining articles were sought for full-text evaluation against the eligibility criteria. All reasons for excluding articles at the full-text stage were carefully recorded and are presented in a PRISMA flow diagram^63^. Study selection and screening were conducted in accordance with the predefined PICO framework. The population included individuals with diabetes undergoing screening for diabetic retinopathy (DR), including any DR, referable DR (RDR), vision-threatening DR (VTDR), and diabetic macular edema (DME). The intervention comprised artificial intelligence (AI)-based diagnostic systems applied to fundus photography for DR screening. The comparator was store-and-forward (SAF) teleophthalmology or conventional remote image-based screening interpreted by human graders. The outcomes of interest were diagnostic accuracy measures, specifically sensitivity and specificity (or data permitting their calculation). All records retrieved from the six databases were independently screened by two reviewers. Titles and abstracts were initially assessed to exclude clearly irrelevant studies. Full-text articles were then reviewed for eligibility. Studies were included if they reported original data on AI-based or SAF screening for DR, used fundus photography as the index test, employed a recognized reference standard (e.g., ICDR, ETDRS, or equivalent), and provided sufficient data to derive sensitivity and/or specificity. Exclusion criteria were non-human studies, reviews, editorials, conference abstracts without full data, studies without a comparator or reference standard, insufficient diagnostic outcome data, and duplicate or overlapping datasets. Disagreements were resolved by consensus. This rigorous PICO-guided process ensured inclusion of clinically relevant and methodologically robust studies for quantitative synthesis.

### Data extraction and risk-of-bias assessment

Data extraction was performed independently by two reviewers using a standardized, pre-piloted extraction form to ensure consistency and accuracy. Extracted variables included study characteristics (author, year, country, study design), population details and sample size, diabetic retinopathy prevalence, imaging protocols, index-test characteristics (AI model or store-and-forward method), reference standards and grading procedures, rates of ungradable images, and diagnostic accuracy outcomes. For each study, data were extracted to derive sensitivity and specificity, including true-positive, false-positive, true-negative, and false-negative values where available. Any discrepancies in extracted data were resolved through discussion and consensus, with re-examination of the original articles when required. In studies evaluating multiple AI algorithms on the same participant cohort or image set, only one algorithm per dataset was included in the primary meta-analysis to avoid unit-of-analysis errors arising from correlated outcomes. The selected algorithm was the model identified by the study authors as the primary or clinically intended system, or the aggregate estimate where reported. Sensitivity analyses were performed using alternative algorithm selections to assess the robustness of pooled estimates.

Quality appraisal was conducted using the QUADAS-2 tool^64^, assessing risk of bias and applicability concerns across four domains: patient selection, index test, reference standard, and flow and timing. Each domain was rated as low risk, some concerns, or high risk based on predefined signaling questions. Applicability was evaluated for patient population, index test, and reference-standard relevance to real-world screening settings. All signaling questions were assessed internally to inform domain-level judgments, with disagreements resolved by consensus. Overall, most studies demonstrated low risk of bias and minimal applicability concerns, supporting the reliability of the pooled diagnostic accuracy estimates presented in this meta-analysis.

### Statistical analysis

All statistical analyses were performed using Comprehensive Meta-Analysis (CMA) software^65^. Diagnostic accuracy outcomes were synthesized separately for AI-based systems and SAF teleophthalmology, and stratified by target condition, including any DR, RDR, VTDR, and DME. Consistent with recommendations from the *Cochrane Handbook for Systematic Reviews of Diagnostic Test Accuracy*, sensitivity and specificity were analyzed separately when substantial clinical and methodological heterogeneity exists across studies, particularly when index-test thresholds, imaging protocols, and case-mix differ between evidence streams. Under such circumstances, pooled operating characteristics may be more interpretable than hierarchical summary ROC modeling when the primary objective is pathway-level implementation interpretation rather than derivation of a single global test threshold. Sensitivity and specificity were analyzed independently to avoid mathematical coupling and to ensure interpretability across heterogeneous study designs. For each outcome, logit-transformed event rates were used as the primary effect size measure, as the logit transformation stabilizes variances and is appropriate for proportions approaching 0 or 1. Pooled estimates were calculated using random-effects models (DerSimonian–Laird method) to account for anticipated clinical and methodological heterogeneity across studies, including differences in populations, imaging protocols, AI architectures, and grading standards. Forest plots were generated for each outcome to visually display individual study estimates, pooled effects, and corresponding 95% confidence intervals. Statistical heterogeneity was assessed using Cochran’s *Q* statistic, *I*², and τ², with *I*² values interpreted according to conventional thresholds. Where heterogeneity was minimal, fixed-effect estimates were additionally reported for comparison. To explore potential sources of heterogeneity and threshold effects, meta-regression analyses were conducted using logit specificity as a covariate to examine its association with logit sensitivity for both AI-based and SAF approaches. Regression coefficients (β), 95% confidence intervals, *Z* values, and p values were reported, along with the proportion of between-study variance explained (*R*² analog). Publication bias was evaluated using funnel plot inspection and formally assessed with Egger’s regression test and Begg’s rank correlation test, where sufficient studies were available. All statistical tests were two-sided, with a significance threshold of *p* < 0.05.

### Clinical utility and decision-analytic translation

In addition to conventional diagnostic accuracy synthesis, pooled sensitivity and specificity estimates were translated into clinically interpretable decision consequences to support implementation-oriented interpretation. Scenario-based analyses were performed to estimate expected screening outcomes per 1000 individuals screened under representative disease prevalence conditions. Three illustrative prevalence scenarios (low, moderate, and high prevalence) were defined based on the range of DR prevalence observed across included studies, allowing estimation of expected true positives, false negatives, false positives, and true negatives for each screening pathway. From these values, referral burden and missed referral-relevant disease per 1000 screened were derived as clinically meaningful decision outcomes.

To avoid reliance on uncertain economic assumptions, the misclassification impact was evaluated using a relative decision-preference framework rather than direct monetary costing. Specifically, sensitivity analyses considered plausible relative weights between false-negative and false-positive classifications (e.g., false-negative to false-positive ratios of 10:1 and 20:1), reflecting the clinical principle that missed referral-relevant disease generally carries substantially greater consequence than unnecessary referral in population screening settings. This framework allowed qualitative assessment of how pathway preference changes under different decision priorities.

Clinical utility was further interpreted using a decision-analytic net-benefit framework consistent with decision-curve methodology. Net benefit was conceptualized as the balance between true-positive detection and false-positive burden weighted by threshold probability, and was interpreted relative to reference strategies including “refer-all” and “refer-none.” Because individual-level risk probabilities were not consistently available across studies, formal patient-level decision-curve analysis was not performed; instead, pooled operating characteristics were translated into test-consequence summaries to provide an implementation-oriented approximation of clinical utility.

In addition, interpretation and reporting of AI-related evidence were aligned conceptually with emerging clinical evaluation frameworks for artificial intelligence in healthcare, including CONSORT-AI and SPIRIT-AI extensions for clinical trial reporting, as well as the DECIDE-AI framework describing early-stage clinical evaluation of AI decision-support systems^66–68^. While these frameworks primarily guide prospective clinical studies rather than meta-analyses, they provide relevant context for interpreting AI performance within real-world workflows, governance structures, and implementation pathways. Accordingly, this review emphasizes pathway-level operating characteristics, workflow integration, and decision consequences rather than algorithm-centric performance claims alone.

## Supplementary information

Supplementary information

Supplementary table

## Data Availability

The datasets generated and/or analyzed during the current study are available from the corresponding author upon reasonable request. All data were extracted from previously published studies, which are cited in the References.

## Code availability

Not applicable. No custom code was generated for this study beyond standard meta-analysis software workflows.
