# Supplementary material for: Clinical setting-dependent diagnostic accuracy of artificial intelligence and store-and-forward diabetic retinopathy screening: a systematic review and meta-analysis
Source: NPJ Digit Med. 2026 May 15;9:400. doi: 10.1038/s41746-026-02627-0 (PMC13212912; doi:10.1038/s41746-026-02627-0)
Supplement: Supplementary file 1 — Supplementary information [file 41746_2026_2627_MOESM1_ESM.pdf]

Supplementary Figures

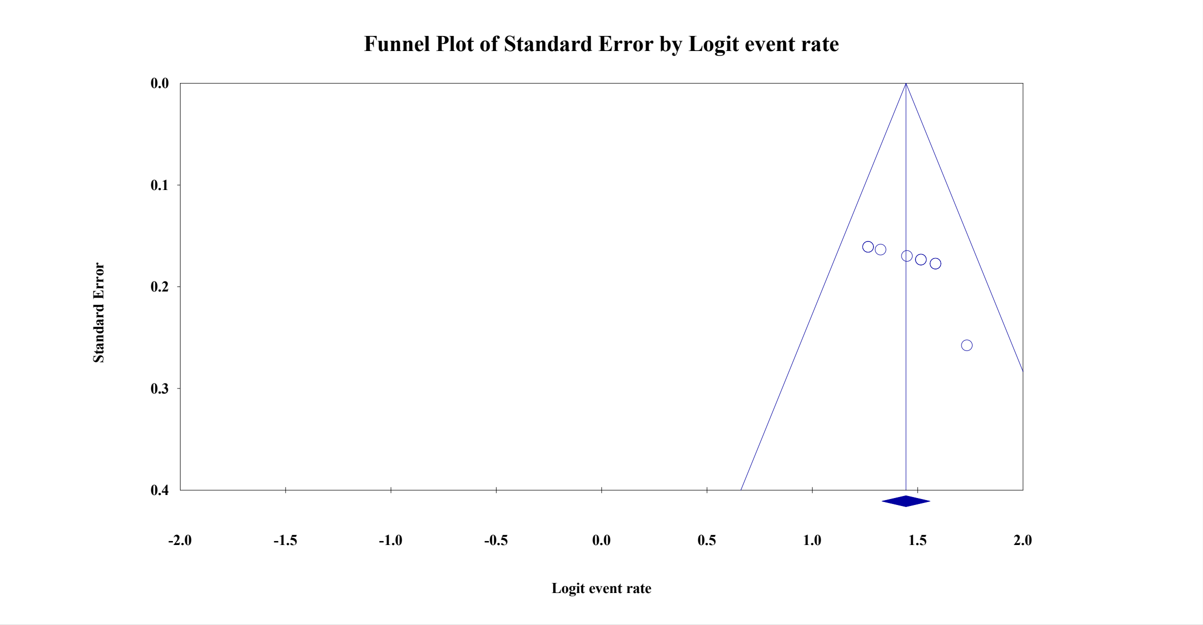

Supplementary Figure 1: Funnel plots for sensitivity of any DR using store-and-forward.

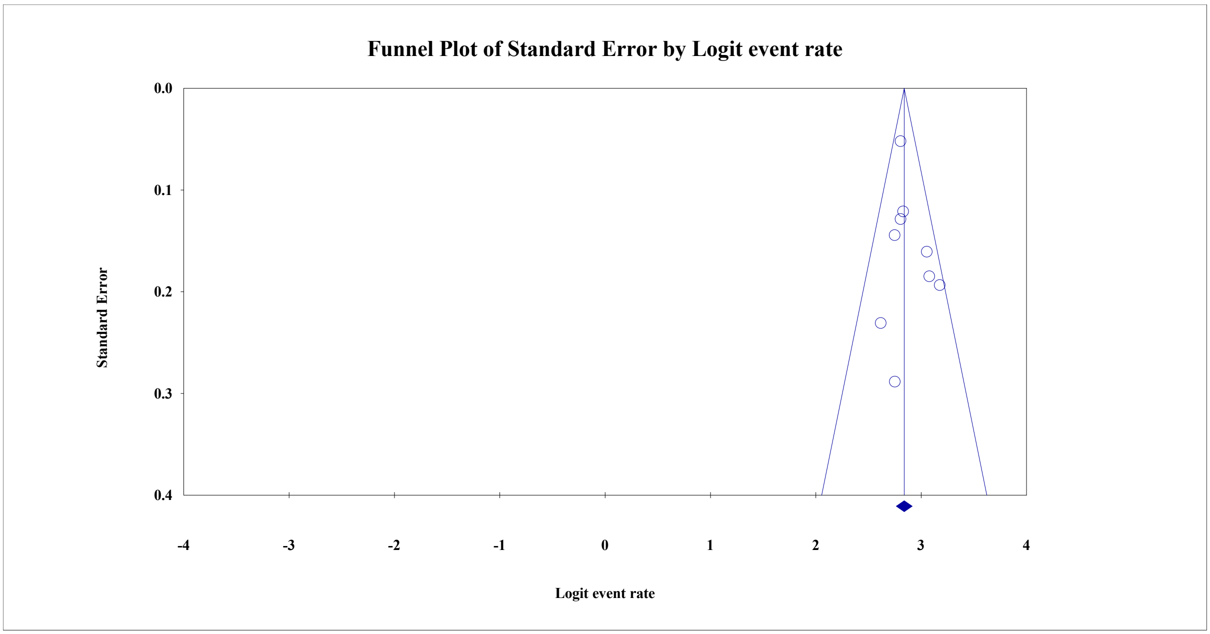

Supplementary Figure 2: Funnel plots for sensitivity of any DR using AI.

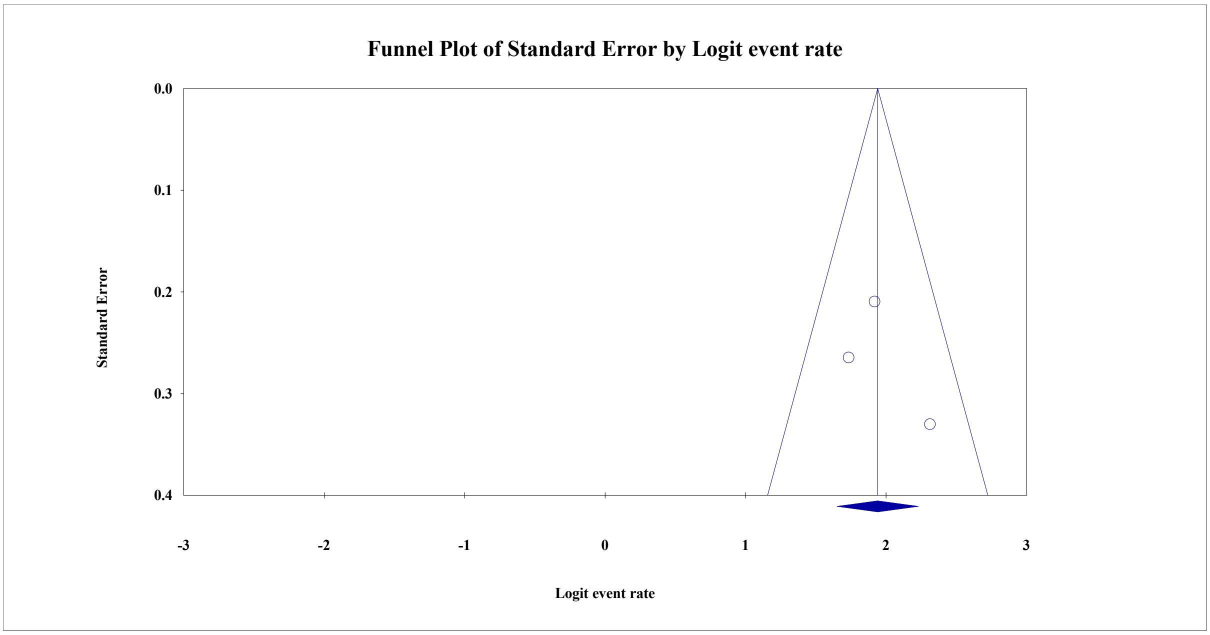

Supplementary Figure 3: Funnel plots for sensitivity of DME using store-and-forward.

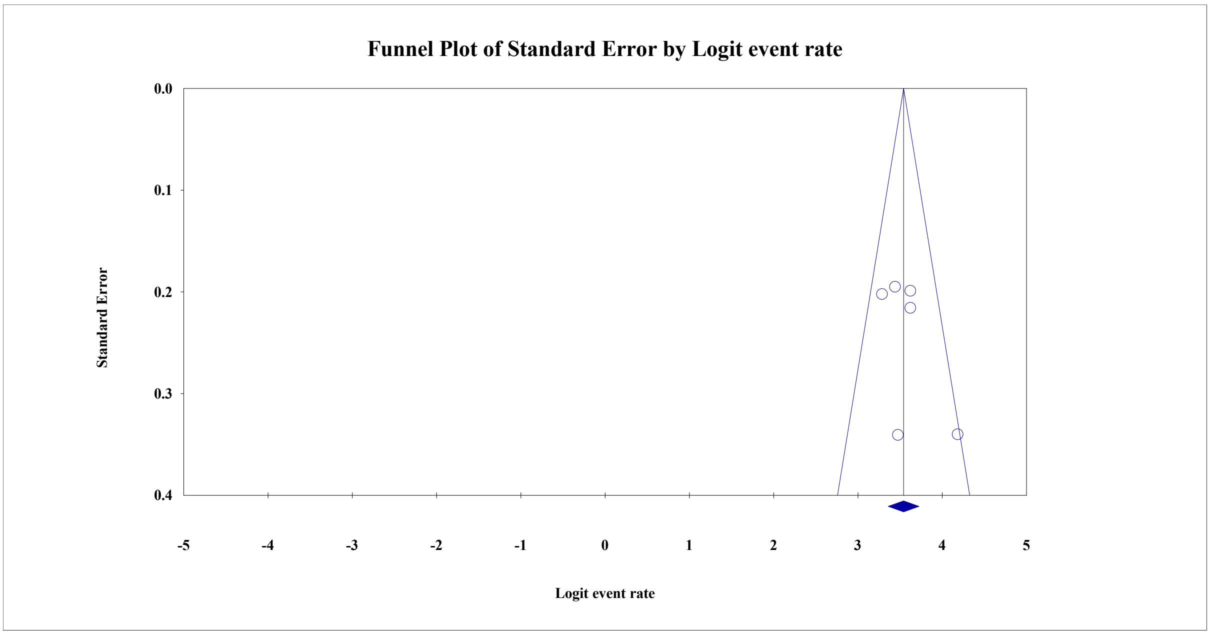

Supplementary Figure 4: Funnel plots for sensitivity of DME using AI.

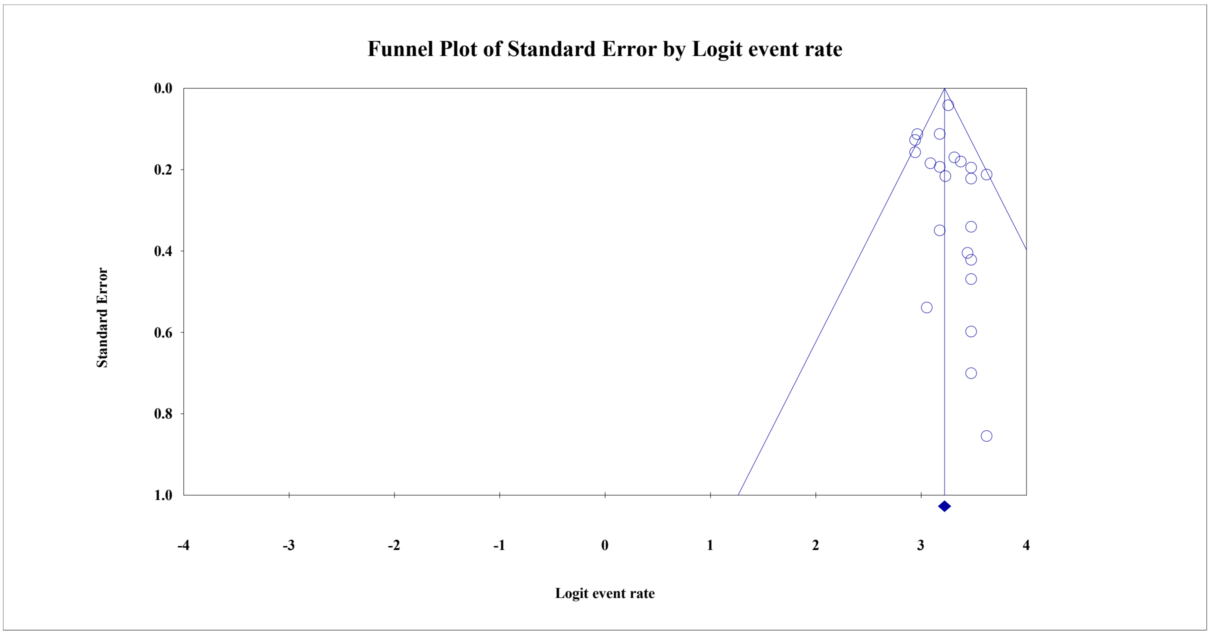

Supplementary Figure 5: Funnel plots for sensitivity of RDR using store-and-forward.

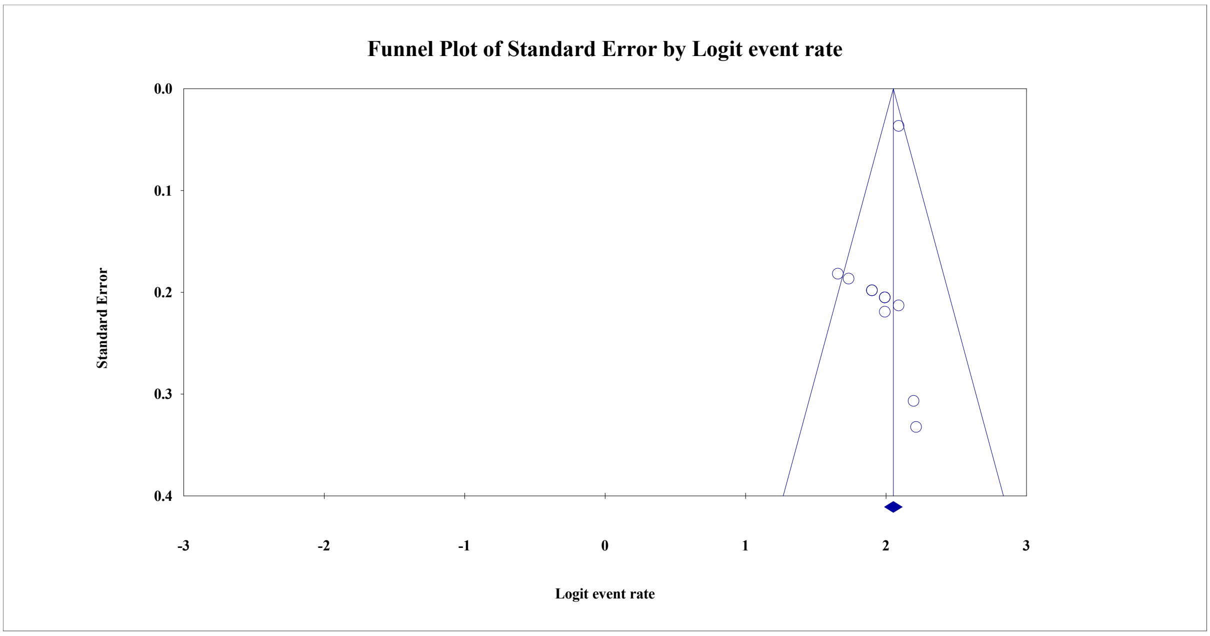

Supplementary Figure 6: Funnel plots for sensitivity of RDR using AI.

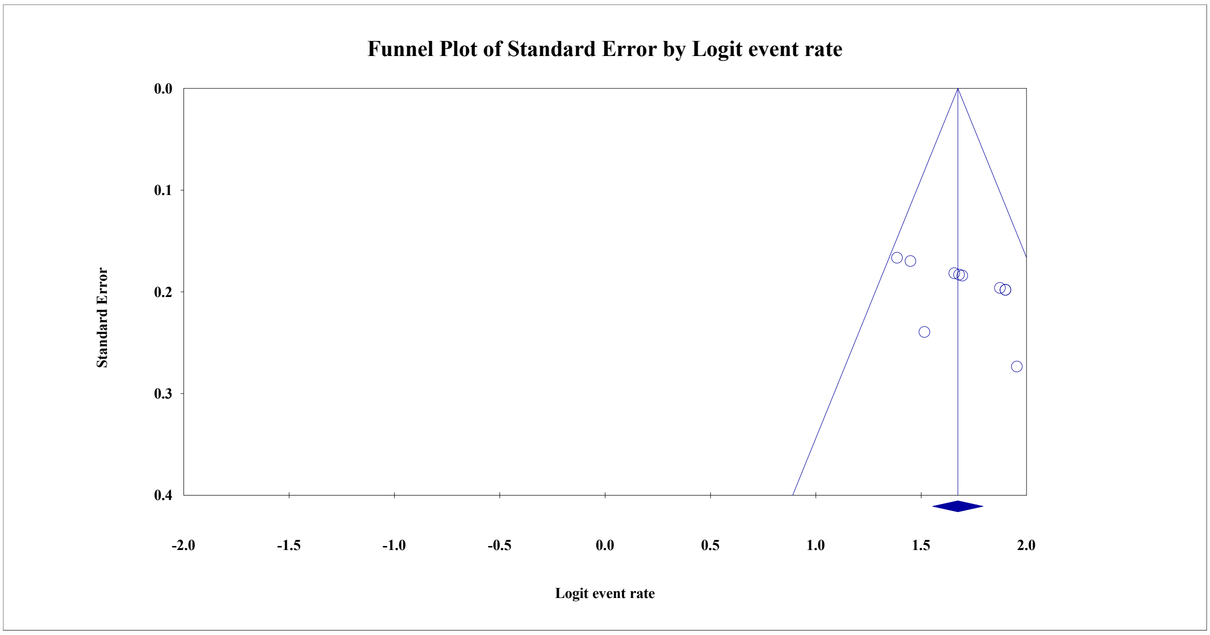

Supplementary Figure 7: Funnel plots for sensitivity of VTDR using store-and-forward.

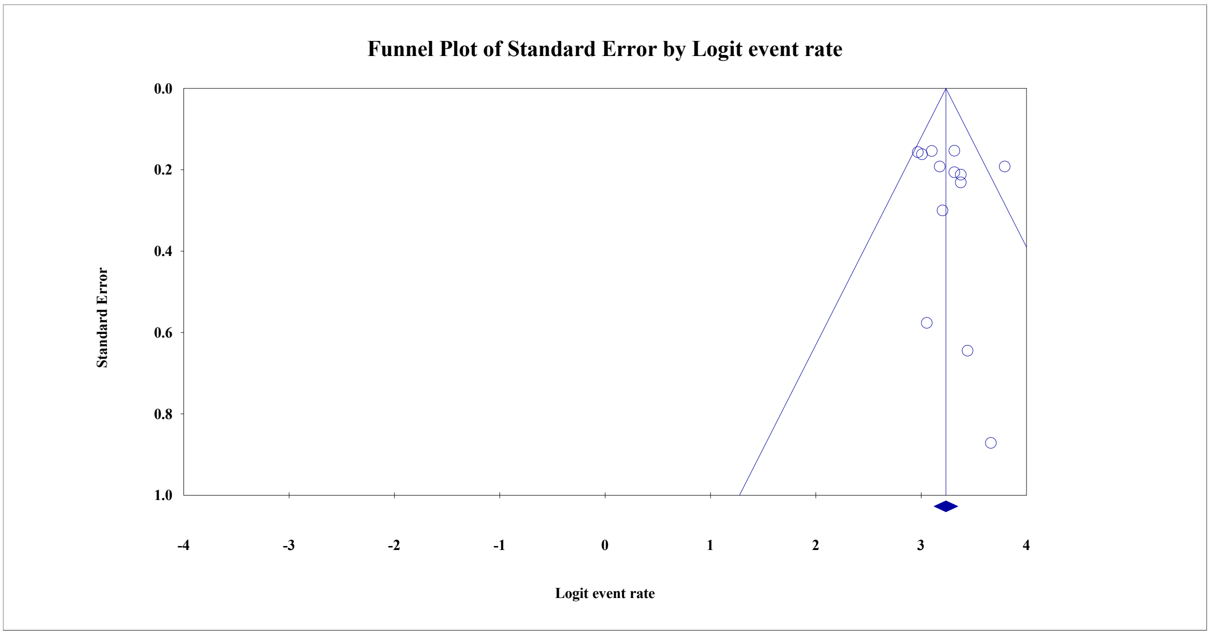

Supplementary Figure 8: Funnel plots for sensitivity of VTDR using AI.

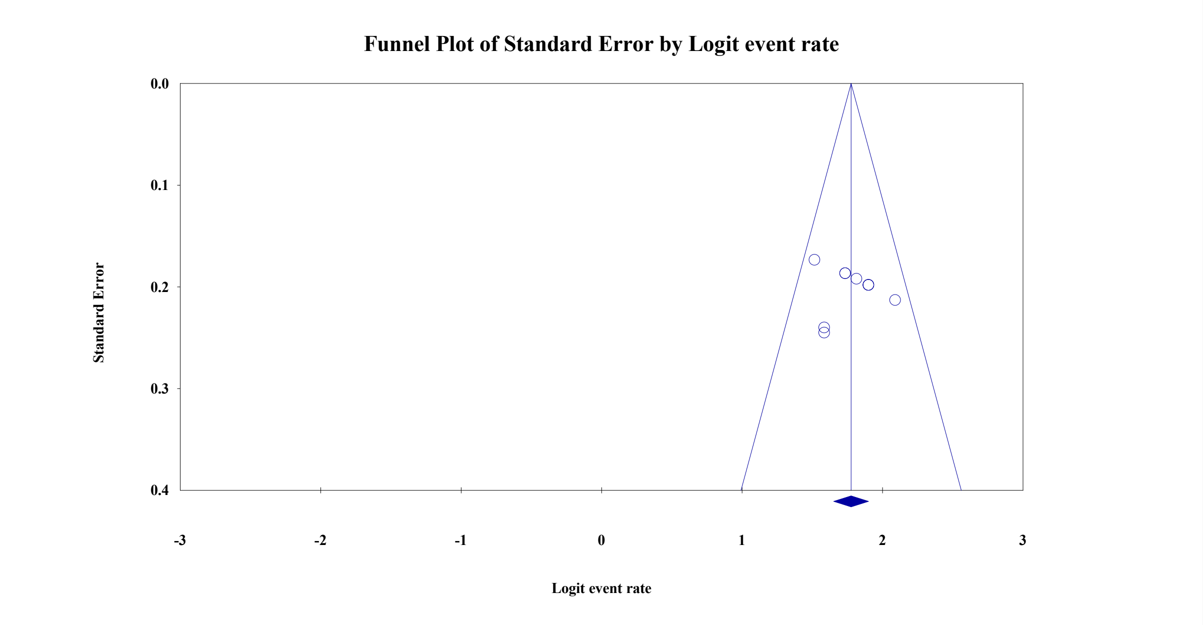

Supplementary Figure 9: Funnel plots for specificity of any DR using store-and-forward.

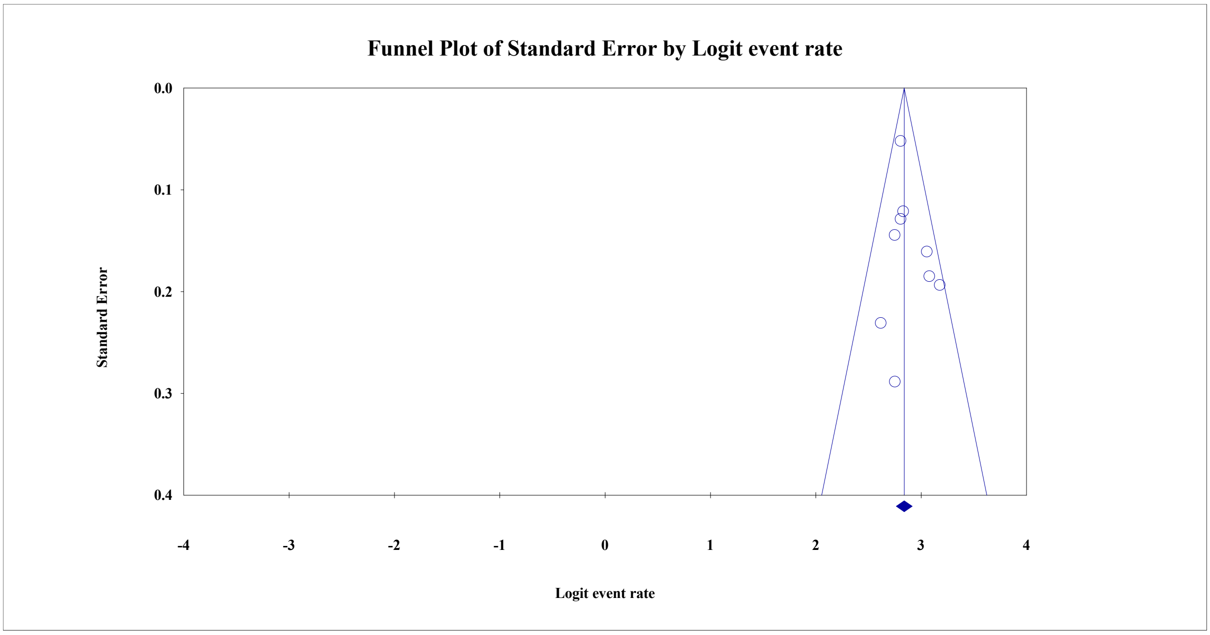

Supplementary Figure 10: Funnel plots for specificity of any DR using AI.

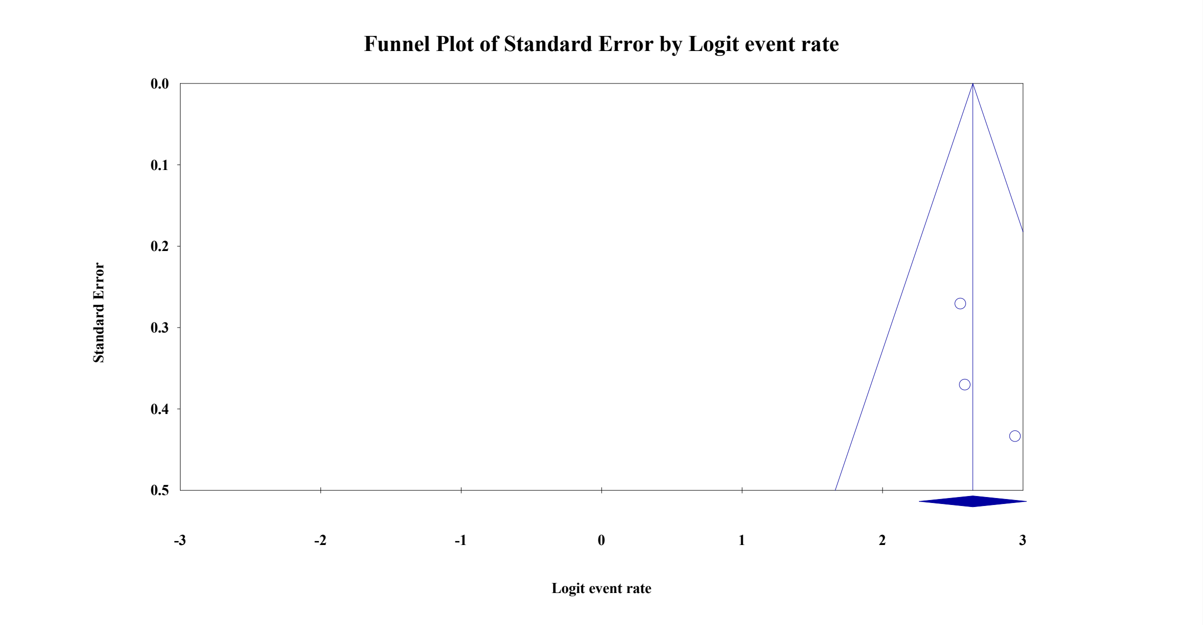

Supplementary Figure 11: Funnel plots for specificity of DME using store-and-forward.

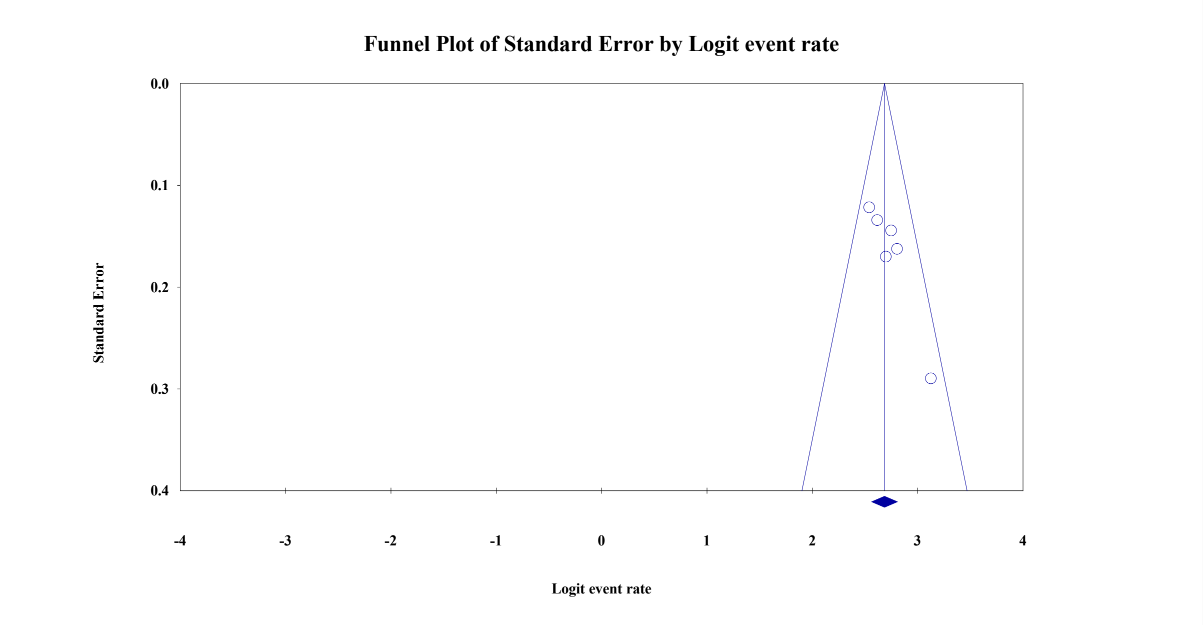

Supplementary Figure 12: Funnel plots for specificity of DME using AI.

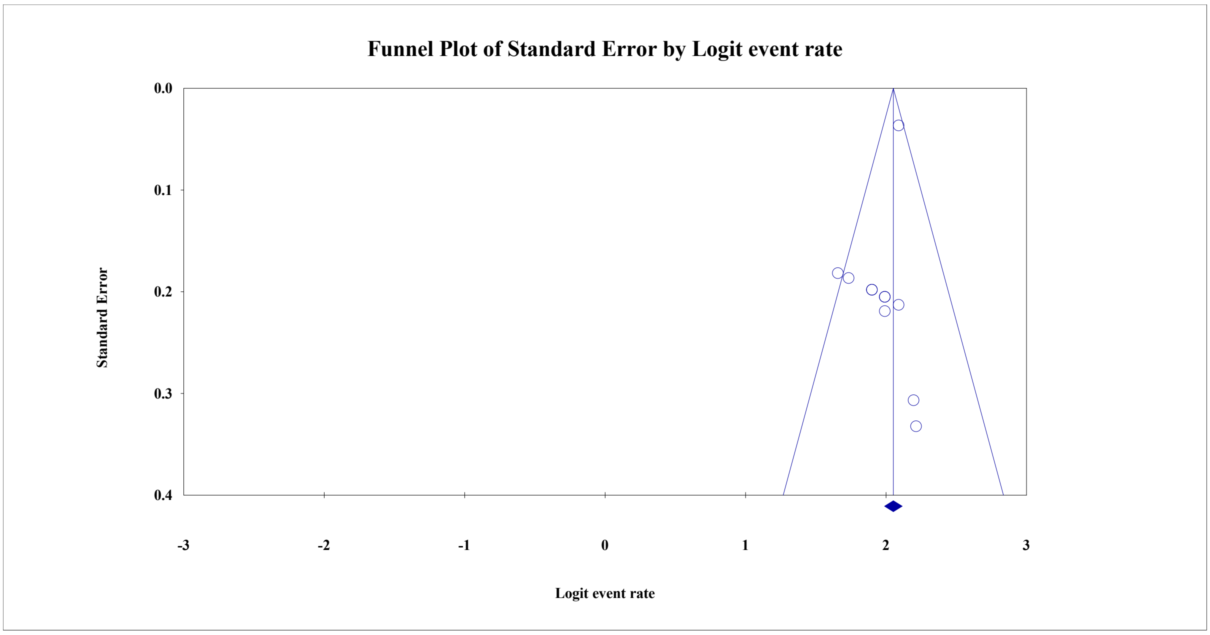

Supplementary Figure 13: Funnel plots for specificity of RDR using store-and-forward.

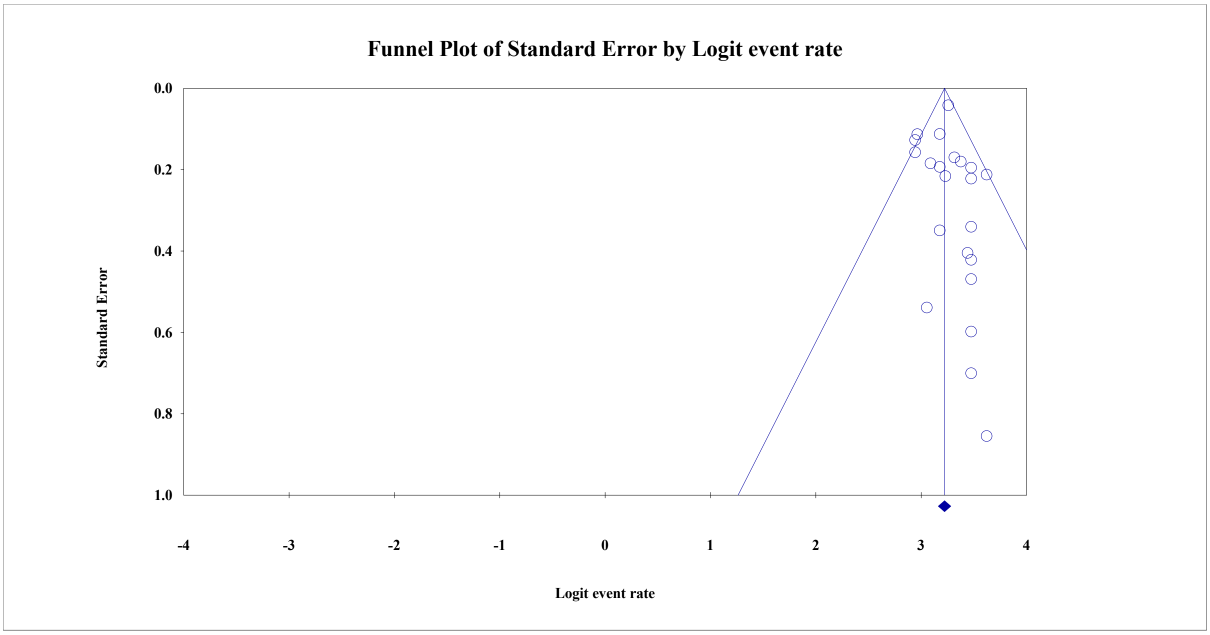

Supplementary Figure 14: Funnel plots for specificity of RDR using AI.

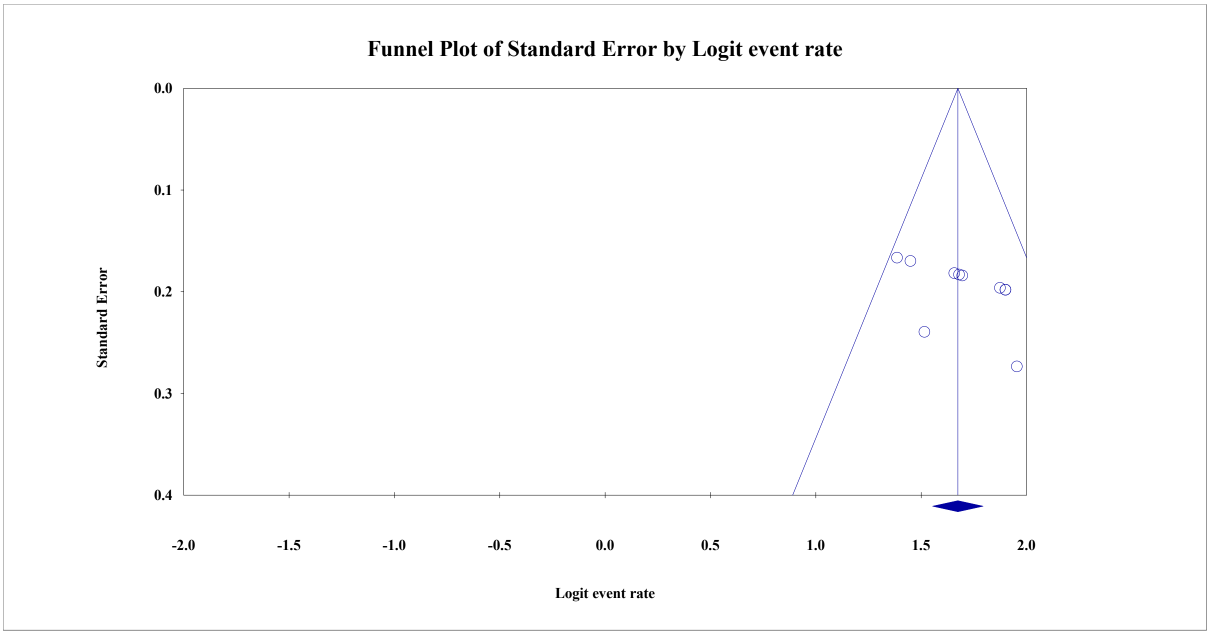

Supplementary Figure 15: Funnel plots for specificity of VTDR using store-and-forward.

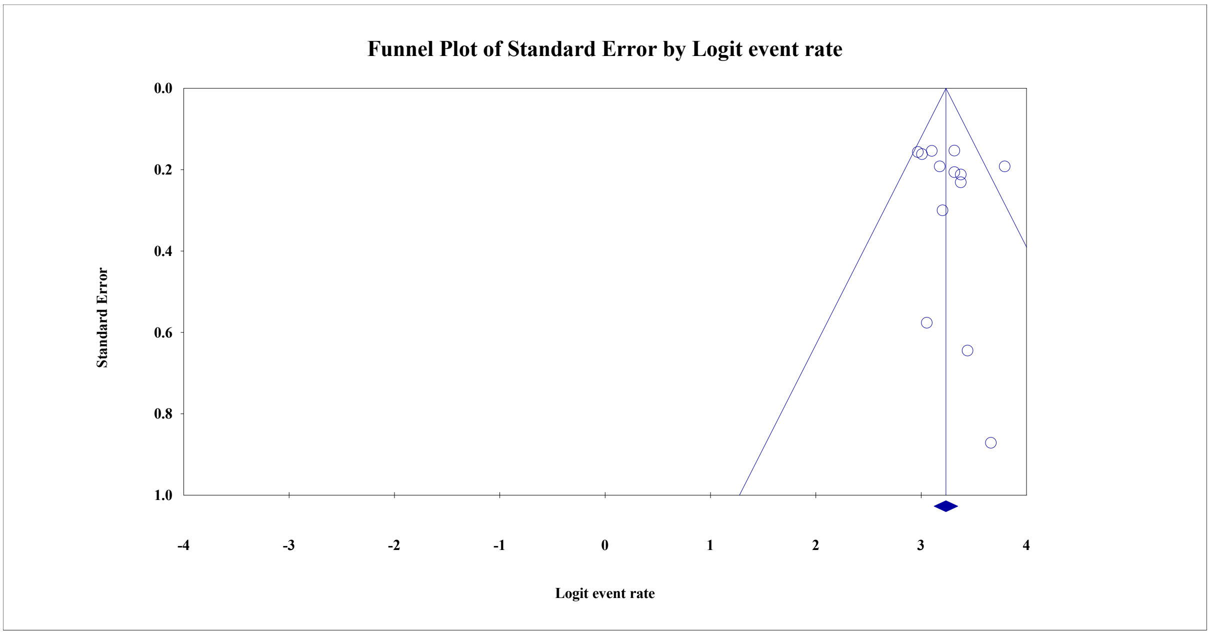

Supplementary Figure 16: Funnel plots for specificity of VTDR using AI.
