# Supplementary material for: Clinical setting-dependent diagnostic accuracy of artificial intelligence and store-and-forward diabetic retinopathy screening: a systematic review and meta-analysis
Source: NPJ Digit Med. 2026 May 15;9:400. doi: 10.1038/s41746-026-02627-0 (PMC13212912; doi:10.1038/s41746-026-02627-0)
Supplement: Supplementary file 2 — Supplementary table [file 41746_2026_2627_MOESM2_ESM.docx]

**Supplementary Table 1. Expected screening consequences per 1,000 screened (RDR and VTDR).**
(*TP = true positives; FN = false negatives; FP = false positives; TN = true negatives. Values are calculated directly from pooled sensitivity/specificity and the specified prevalence.*)

**A) Referable DR (RDR)**

| **Prevalence of RDR** | **Pathway** | **TP** | **FN** | **FP** | **TN** |
| --- | --- | --- | --- | --- | --- |
| 5% | AI (Se 96.2%, Sp 96.2%) | 48 | 2 | 36 | 914 |
| 5% | SAF (Se 88.6%, Sp 87.2%) | 44 | 6 | 122 | 828 |
| 15% | AI (Se 96.2%, Sp 96.2%) | 144 | 6 | 32 | 818 |
| 15% | SAF (Se 88.6%, Sp 87.2%) | 133 | 17 | 109 | 741 |
| 30% | AI (Se 96.2%, Sp 96.2%) | 289 | 11 | 27 | 673 |
| 30% | SAF (Se 88.6%, Sp 87.2%) | 266 | 34 | 90 | 610 |

**B) Vision-threatening DR (VTDR)**

| **Prevalence of VTDR** | **Pathway** | **TP** | **FN** | **FP** | **TN** |
| --- | --- | --- | --- | --- | --- |
| 5% | AI (Se 96.2%, Sp 96.4%) | 48 | 2 | 34 | 916 |
| 5% | SAF (Se 84.2%, Sp 90.5%) | 42 | 8 | 90 | 860 |
| 15% | AI (Se 96.2%, Sp 96.4%) | 144 | 6 | 31 | 819 |
| 15% | SAF (Se 84.2%, Sp 90.5%) | 126 | 24 | 81 | 769 |
| 30% | AI (Se 96.2%, Sp 96.4%) | 289 | 11 | 25 | 675 |
| 30% | SAF (Se 84.2%, Sp 90.5%) | 253 | 47 | 66 | 634 |

Interpretation: across plausible prevalence ranges, both pathways provide clinically useful discrimination, but they generate different consequence profiles. For referral-relevant endpoints (RDR/VTDR), AI yields fewer missed cases (FN) at the cost of variable FP differences depending on endpoint and setting, whereas SAF yields higher FP in several RDR scenarios due to lower pooled specificity. These absolute differences are the quantities that determine specialist workload and preventable missed disease in implementation.
